# Bilateral symmetry of linear streptomycete chromosomes

**DOI:** 10.1099/mgen.0.000692

**Published:** 2021-11-15

**Authors:** Lis Algora-Gallardo, Jana K. Schniete, David R. Mark, Iain S. Hunter, Paul R. Herron

**Affiliations:** ^1^​ Strathclyde Institute of Pharmacy and Biomedical Sciences, University of Strathclyde, Glasgow G4 0RE, UK; ^2^​ Department of Biology, Edge Hill University, Ormskirk L39 4QP, UK

**Keywords:** bilateral symmetry, linear chromosome, *oriC*, *Streptomyces*

## Abstract

Here, we characterize an uncommon set of telomeres from *Streptomyces rimosus* ATCC 10970, the parental strain of a lineage of one of the earliest-discovered antibiotic producers. Following the closure of its genome sequence, we compared unusual telomeres from this organism with the other five classes of replicon ends found amongst streptomycetes. Closed replicons of streptomycete chromosomes were organized with respect to their phylogeny and physical orientation, which demonstrated that different telomeres were not associated with particular clades and are likely shared amongst different strains by plasmid-driven horizontal gene transfer. Furthermore, we identified a ~50 kb origin island with conserved synteny that is located at the core of all streptomycete chromosomes and forms an axis around which symmetrical chromosome inversions can take place. Despite this chromosomal bilateral symmetry, a bias in *parS* sites to the right of *oriC* is maintained across the family *Streptomycetaceae* and suggests that the formation of ParB/*parS* nucleoprotein complexes on the right replichore is a conserved feature in streptomycetes. Consequently, our studies reveal novel features of linear bacterial replicons that, through their manipulation, may lead to improvements in growth and productivity of this important industrial group of bacteria.

## Data Summary

Supplementary data files are available from Figshare: https://doi.org/10.6084/m9.figshare.16553307 [1]. Sequence data for the *Streptomyces rimosus* ATCC 10970 chromosome and plasmid are deposited with the National Center for Biotechnology Information (NCBI) under accession numbers NZ_CP048261.1 and NZ_CP048262.1, respectively (BioSample SAMN02471950; BioProject PRJNA182749). The location and identities of all putative *parS* sites across all 20 closed streptomycete genome sequences are provided in Supplementary File S1 (available with the online version of this article and via Figshare).

**Impact Statement**

After closing the genome sequence of *Streptomyces rimosus *, one of the earliest-discovered antibiotic-producing bacteria, we identified an uncommon set of chromosome and plasmid ends. Unusually amongst bacteria, streptomycetes have linear chromosomes and further analysis of closed streptomycete genomes allowed us to bring together a description of the known telomeres found in this family for the first time. We also identified that different ends were not associated with particular groups and are likely shared amongst different strains by plasmid-driven horizontal gene transfer, potentially responsible for the evolution of the great metabolic diversity displayed by this bacterial group. Furthermore, we identified a genomic island located at the core of streptomycete chromosomes that forms an axis around which symmetrical chromosome inversions can take place. Despite this bilateral symmetry, a bias in *parS* sites to the right of the replication origin is maintained across the streptomycetes and suggests that the formation of ParB/*parS* nucleoprotein complexes on the right-hand arm of the chromosome is a conserved feature in streptomycetes. Consequently, our studies reveal novel features of streptomycete replicons that expand our understanding of genomic rearrangements in bacteria that may lead to improvements in the productivity of this important industrial group of bacteria.

## Introduction

**Fig. 1.**
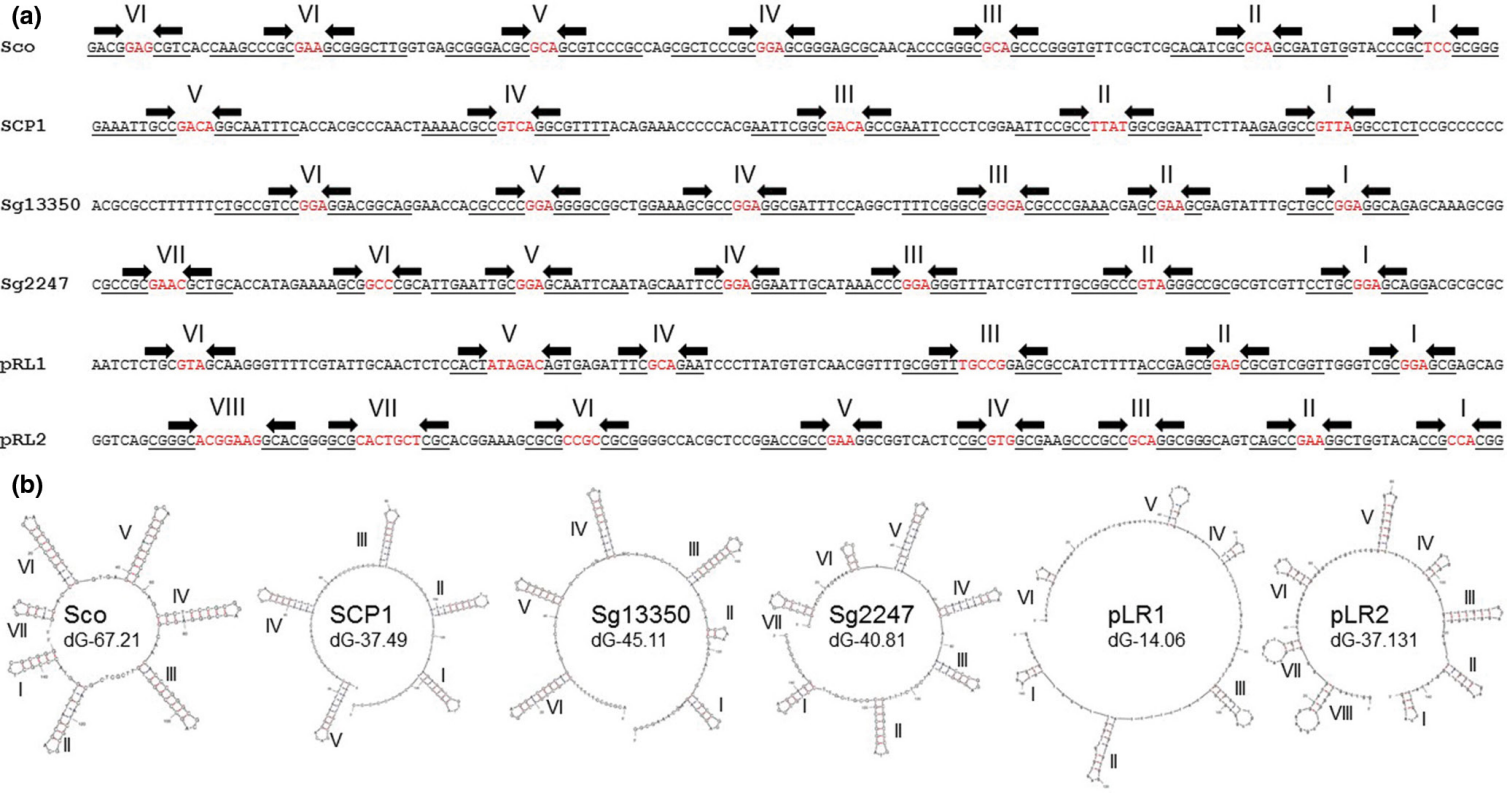
The six known classes of streptomycete telomeres. (**a**) Terminal 150 bp of 3′ replicon ends of known streptomycete telomeres showing stem structures (underlined, arrows) and hairpins (red) identified using Mfold (I–VIII). Sco, archetypal end from *S. coelicolor* chromosome [25]; SCP1, non-archetypal end from *S. coelicolor* plasmid SCP1 [15]; Sg13350, chromosome end from *S. griseus* 13350 chromosome [13]; Sg2247, chromosome end from *S. rimosus* ATCC 10970 chromosome (this work); pRL1, end from *Streptomyces* sp. 44030 plasmid pRL1 [12]; pRL2, end from *Streptomyce* s sp. 440414 plasmid pRL2 [12]. (**b**) Mfold projections of the terminal 150 bp of the known classes of streptomycete telomeres showing stem-loop structures (I–VIII) identified using Mfold [28] are displayed, where the revised free energies (ΔG) were determined using Jacobson–Stockmeyer theory to assign free energies to multi-branch loops. Further details of known streptomycete telomeres are displayed in Table S2 and each individual Mfold representation in Fig. S4.

Streptomycetes are important industrial and environmental bacteria with large linear chromosomes of up to 12 Mb in size [2]. They are renowned for their extensive metabolic repertoire, producing metabolites used in healthcare and industry [3]. The bacteria undergo a complex life cycle involving germination from dormant spores, vegetative growth, differentiation and sporulation again, whilst production of the useful metabolites is intrinsically linked with the developmental life cycle [4]. With this as background, we undertook an analysis of the genome of *Streptomyces rimosus *, the commercial producer of oxytetracycline. Accurate prediction of the molecules produced by these organisms is dependent on the presence of a complete genome sequence; in the case of organisms with linear chromosomes, knowledge of the telomeres of individual replicons is essential to delineate the full coding capacity of the genome and also to gain understanding of its basic architecture. Following the closure of the *S. rimosus* genome sequence, we noticed that the locations of certain genes and sequence motifs required for effective replication and segregation of its genetic material were shared with other streptomycetes. Bidirectional DNA replication in most bacteria begins at a central origin (*oriC*) and proceeds around the chromosome until the two replication forks meet at the terminus (*ter*) of the circular molecule [5]. Streptomycetes possess linear chromosomes and plasmids [6] flanked by terminal inverted repeats (TIRs) that can be over 600 kb in size [7] and end in telomeres that can form complex stem-loop structures [8]. Here, we use the term streptomycete to refer to members of the three genera that form the family *Streptomycetacea* e: *Streptomyces *, *Kitasatospora* and *Streptacidiphilus* [9]. Archetypal chromosome ends (telomeres) are the best characterized termini of streptomycete linear replicons and contain several palindromes with differences in sequence that occur in complementary pairs of stem structures in the terminal 150 bp [8]. These palindromes adopt a clover-leaf structure required for priming by palindrome I in archetypal telomeres [10]. However, there are also non-archetypal telomeres, such as those of SCP1, a giant linear plasmid (GLP) of *Streptomyces coelicolor *. In addition, a number of other non-archetypal telomeres have been identified, such as those from the chromosomes of *Streptomyces griseus* 2247, *S. griseus* 13350 and two linear plasmids, pLR1 and pLR2 [11–13]. Whist all known telomeres contain palindromes, their sequences differ between each class (Fig. 1).

While end-patching of archetypal streptomycete telomeres is well understood and is carried out by the telomeric proteins, Tap and Tpg [10], there is still a gap in our knowledge for most other types of telomeres. *tap* and *tpg* are essential for the end-patching of linear streptomycete replicons with archetypal telomeres, as their deletion leads to replicon circularization [14]. Tac and Tpc are required for end-patching of SCP1 telomeres [15], but there is no mechanistic information for end-patching of other non-archetypal streptomycete telomeres.

The main aim of this research was to complete the genome sequence of *S. rimosus* ATCC 10970 [16, 17]. Key to this was to determine the telomeric sequences of the two *S. rimosus* replicons: a chromosome and GLP, first identified by PFGE [18, 19]. However, when attempting a comparison with other streptomycete genomes, we were only able to identify a small number of streptomycete genome sequences flanked by known telomeres and, thus, truly closed. We then set out to analyse these closed sequences for the location of *oriC*, *parS* sites and genes encoding the telomeric proteins (Tap/Tpg) and partitioning proteins (ParA/ParB) [20, 21]. In so doing, we identified shared features of streptomycete linear chromosomes that increase our understanding of how genomic architecture is conserved in this important antibiotic-producing bacterial group.

## Methods

### Genomic DNA extraction, sequencing and genome assembly

*S. rimosus* was obtained from Pfizer [16] and propagated on Emerson’s agar at 30 °C (4 g beef extract l^−1^, 1 g yeast extract l^−1^, 4 g peptone l^−1^, 10 g glucose l^−1^, 2.5 g NaCl l^−1^, 20 g agar l^−1^, pH 7.2) before inoculating Tryptone Soya Broth (Oxoid) [22], incubated at 30 °C, 250 r.p.m. in 250 ml baffled flasks for 36 h prior to genomic DNA isolation or preparation of PFGE plugs. For the former, biomass was harvested by centrifugation and resuspended in 300 µl TE buffer, pH 7.5, with 10 mg lysozyme ml^−1^ and RNase (0.1 mg ml^−1^) and incubated for 90 min at 37 °C. A 50 µl aliquot of 10% (w/v) SDS was added and the sample mixed, followed by addition of 85 µl 5 M NaCl. A total of 400 µl phenol/chloroform/isoamyl alcohol (25:24:1) was added, vortexed for 30 s and centrifuged for 10 min at 8000 r.p.m. The aqueous layer was added to a new tube and the previous two steps were repeated. A total of 400 µl chloroform/isoamyl alcohol (24:1) were added and vortexed for 30 s and centrifuged for 10 min at 8000 r.p.m. The aqueous layer was added to a new tube and 0.5 ml isopropanol added. The tube was inverted and incubated for 5 min at room temperature. DNA was pelleted at 10 000 r.p.m., washed with 70% (v/v) ethanol, the supernatant removed and the pellet air dried for 30 min at room temperature. The DNA was resuspended in 10 mM Tris buffer pH 7.5 and sent for sequencing by PacBio at NU-OMICS (https://www.northumbria.ac.uk/business-services/engage-with-us/research/nu-omics/) and by Illumina 2×250 bp paired-end reads at MicrobesNG (https://microbesng.com/). DNA was quantified using the Qubit dsDNA HS assay. Details of sequence assembly are provided in the Supplementary Information.

### Determination of telomere sequences

Purification of the telomeric sequences was performed following an adaptation of a previously described procedure [23]. A total of 1 µg genomic DNA of *S. rimosus* was digested overnight with the blunt-end restriction enzymes SmaI and PvuII. The products were then purified using the Promega PCR Clean-Up kit and eluted in a final volume of 90 µl with dH_2_O. A total of 10 µl 1 M NaOH was added and the mixture was incubated for 1 h at 37 °C. The samples were neutralized using 2 M HCl and 1 M Tris (pH 8) was added to a final concentration of 0.1 M. A 20× SSC solution was then added to final concentration of 2× and the samples were incubated at 68 °C for 1 h. The samples were purified once again with the Promega PCR Clean-Up kit and ligated overnight using Promega T4 DNA ligase. Inverted PCR analyses were performed using the primers listed in Table S1, and the amplified products were purified and sequenced by Eurofins Genomics.

### PFGE

PFGE analyses were performed using the Bio-Rad CHEF-DR II PFGE system. Agarose plugs containing DNA were prepared from TSB grown liquid cultures of *S. rimosus* using established procedures [22], with the following amendments to reduce DNA degradation [24]. HEPES was substituted for Tris in buffers and mycelium was washed in HES buffer (25 mM HEPES-NaOH, 25 mM EDTA, 0.3 M sucrose, pH 8) and digested in 1 mg lysozyme ml^−1^ in HES buffer. After lysis, plugs were washed three times with NDS (1% *N*-laurylsarcosine, 0.5M EDTA, 10 mM glycine, pH 9.5). The plugs were incubated overnight with NDS containing 1 mg proteinase K ml^−1^ and 1 mM CaCl_2_. The next day, the plugs were washed in HE buffer (10 mM HEPES-NaOH, 1 mM EDTA, pH 8). After 15 min at room temperature, the liquid was replaced with fresh buffer and 1 µl BSA (10 µg ml^-1^), and 50 U AseI or DraI added. The mix was incubated overnight at 37 °C. In the case of AseI digestion, a second sample of 10 U enzyme was added after 2 h. For electrophoresis, HEPES buffer (16 mM HEPES-NaOH, 16 mM sodium acetate, 0.8 mM EDTA, pH7.5) was used and the voltage set at 4 V cm^−1^, with an initial switch time of 70 s and a final switch time of 130 s. Electrophoresis was performed for 24 h. The gel was then stained for 30 min in 1 µg ethidium bromide ml^−1^ solution and de-stained with dH_2_O for an hour. The gel was imaged with a UV trans-illuminator.

### Telomere identification and analysis

In order to identify streptomycete telomeres, we used the terminal 36 and 150 bp of the six classes of telomeres to search the National Center for Biotechnology Information (NCBI) database using blastn. The two sizes of query sequences were taken from: the *S. coelicolor* chromosome end (Sco) [25]; the end of *S. coelicolor* plasmid SCP1 (SCP1) [15]; the chromosome end of *S. griseus* 13350 (Sg13350) [13]; the chromosome end of *S. rimosus* ATCC 10970 (Sg2247) (this work); the end of *Streptomyces* sp. 44030 plasmid pRL1 (pRL1) [12]; the end of *Streptomyce* s sp. 440414 plasmid pRL2 (pRL2) [12]. The query sequences are described in Fig. 1. Only those significant hits from complete whole-genome sequencing projects that were either located at the end of a replicon or where sequences were physically recovered and sequenced were designated as telomeres.

A ClustalW alignment of 13 Sg2247 independent telomeres was used to map telomere evolutionary history and was inferred using the maximum-likelihood method and the Tamura three-parameter model [26]. The tree with the highest log likelihood (−1238.57) was used. Initial tree(s) for the heuristic search were obtained automatically by applying Neighbor-Join and BioNJ algorithms to a matrix of pairwise distances estimated using the maximum composite likelihood (MCL) approach, and then selecting the topology with superior log likelihood value. A discrete gamma distribution was used to model evolutionary rate differences among sites [five categories (+G, parameter=0.6336)]. Trees were drawn to scale, with branch lengths measured by the number of substitutions per site. This analysis involved 13 nucleotide sequences and there were 163 positions in total in the final dataset. Evolutionary analyses were conducted in mega x [27].

Stem-loop structures identified using Mfold [28] are displayed, where the revised free energies (ΔG, kJ mol^–1^) were determined using Jacobson–Stockmeyer theory to assign free energies to multi-branch loops. Projections were calculated using default conditions except folding temperature was set at 30 °C, Na^+^ concentration 0.05 M and maximum distance between paired bases was set at 20 [10]. Closed chromosome sequences from the genus *Streptomyces *, in conjunction with the closed sequence of *Kitasatospora setae* KM-6054 as an outgroup, were used to carry out multilocus sequence analysis (MLSA) to produce a high-resolution species tree using AutoMLST after a concatenated alignment [29]. The average nucleotide identities between 20 closed streptomycete genomes were calculated using the OrthoANI tool [30] and plotted with the heat map function in R (ver. 4.0).

Closed streptomycete chromosomes were subjected to a progressive alignment in Mauve [31] using default settings and dot plots generated by Nucmer (Galaxy version 4.0.0beta2 +galaxy0) [32] with default settings, except that the filter setting was turned on so only delta alignments which represent the 'best' hit to any particular location on either sequence were displayed. Maps showing synteny or origin islands were created using clinker and clustermap.js [33].

The locations of predicted *parS* sites were determined by searching the 20 closed genomes using the consensus matrix for bacterial *parS* sites [34]. This was done by performing a ClustalW alignment of the 1030 predicted *parS* sited previously identified [34]. A consensus matrix was then constructed in WebLogo 3 (Galaxy version 4.0.0beta2 +galaxy0) [35] and used to interrogate closed streptomycete genomes using matrix scan in the Regulatory Sequence Analysis Tools suite (rsat) (http://embnet.ccg.unam.mx/rsat//matrix-scan-quick_form.cgi) [36]. For this analysis the threshold weight score was set at >15 [34].

## Results and Discussion

### Closed genome of *S. rimosus* ATCC 10970 consists of two linear replicons

*S. rimosus* is one of the most sequenced streptomycetes with 38 records listed in the NCBI database at time of writing (assembly level: complete, 2; chromosome, 1; scaffold, 2; contig, 33), although none contain recognizable streptomycete telomeres. Analysis of 32 *S *. *rimosus* genome sequences shows that members of this species encode between 35 and 71 specialized metabolite biosynthetic gene clusters [37]. Many streptomycetes contain specialised metabolite biosynthetic gene clusters in the arms of linear chromosomes [38], whose annotation is likely complicated by the presence of multiple contigs and TIRs. Consequently, we used a combination of PacBio and Illumina sequencing, in conjunction with the physical recovery and sequencing of the chromosome and plasmid ends, to generate a fully closed sequence of *S. rimosus *. The genome assembly details are described in the Supplementary Information.

The genome of *S. rimosus* ATCC 10970 consists of a linear chromosome and plasmid, SRP1 (Fig. S1a), which is consistent with earlier physical studies [18, 19]. The chromosome is 9 351 267 bp in size with 11 386 bp TIRs. The GLP, SRP1, is 292 624 bp in size with TIRs of 288 bp. Together these two replicons encode 8292 coding sequences, 7 rRNA operons, 68 tRNAs and 3 non-coding RNAs. The *S. rimosus* ATCC 10970 genome is listed in the NCBI database under accession numbers NZ_CP048261.1 (chromosome) and NZ_CP048262.1 (plasmid). The size of the GLP, SRP1, was verified by PFGE and the assemblies of both replicons were verified by DraI and AseI restriction digestion followed by PFGE (Fig. S1b). The restriction patterns observed agreed with the *in silico* predictions of their size and corroborates our assembly. PFGE patterns from *S. rimosus* R7 (R7 and G7 are synonyms for ATCC 10970) were similar to those from *S. rimosus* R6 [19] except that an additional fragment was located at one chromosome end of R7. This suggests R6 and R7 share a common ancestor and might account for the different telomeres found in *S. rimosus* R6 and R7 [16] and reported here.

### Replicons of *S. rimosus* ATCC 10970 are flanked by rare telomeres

**Fig. 2.**
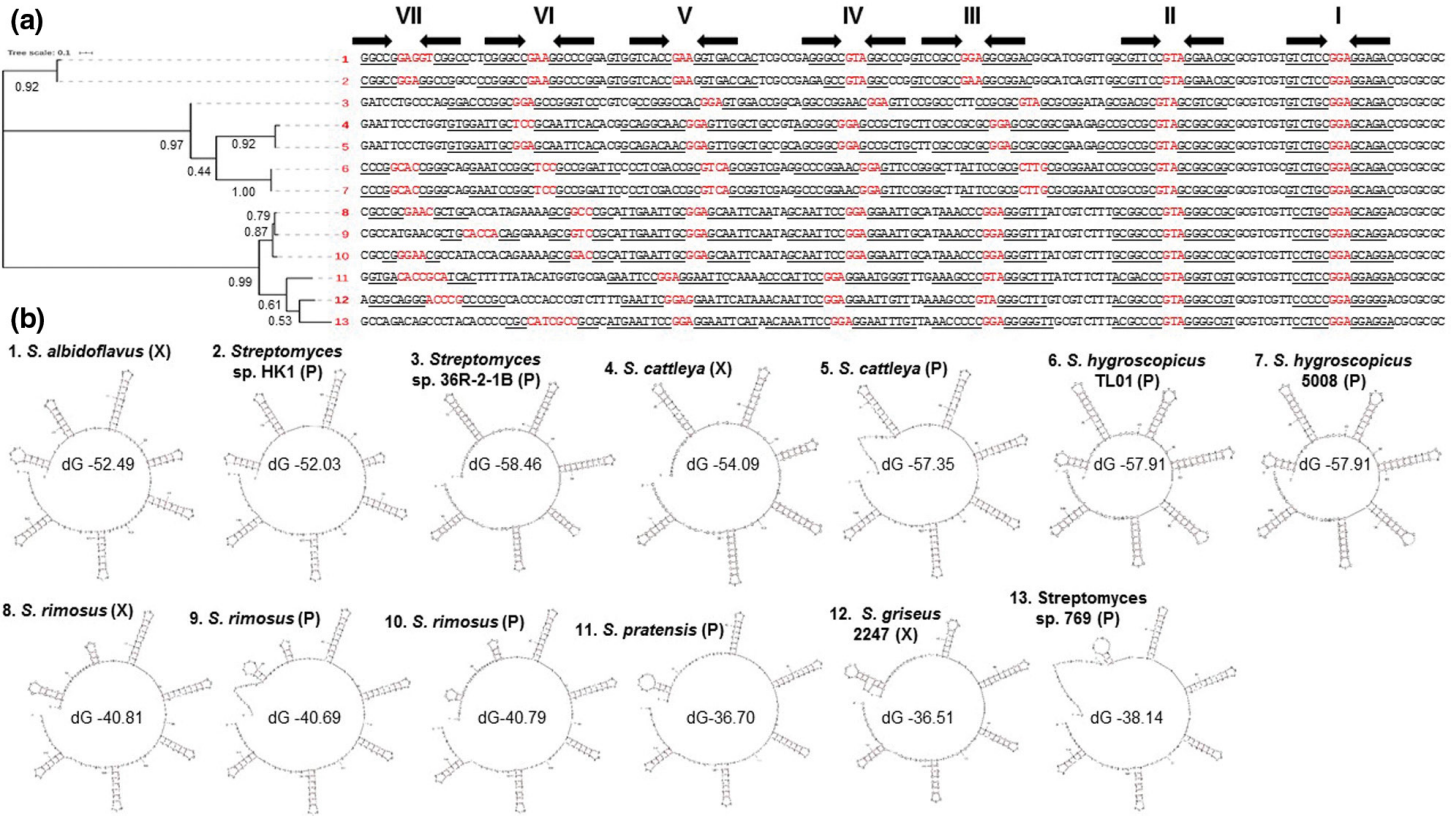
Evolutionary relationships of Sg2247-type telomeres showing stem-loop structures. (**a**) Phylogenetic tree of 3′ replicon end sequences with similarity to the telomeres of *S. rimosus* ATCC 10970 (Sg2247-type telomeres) [11]. The proportion of trees in which the associated taxa clustered together is shown next to the branches. The tree is drawn to scale, with branch lengths measured in the number of substitutions per site. 1, *S. albidoflavus* J1074 chromosome, accession number NC_020990.1; 2, *Streptomyces* sp. HK1 plasmid pSHK1, EU372836.1; 3, *Streptomyces* sp. 36R-2-1B plasmid pYY8L, GU080325.1; 4, ' *S. cattleya* ' NRRL 8057 chromosome, FQ859185.1; 5, ' *S. cattleya* ' NRRL 8057 plasmid pSCAT, FQ859184.1; 6, *S. hygroscopicus* subsp. *jinggangensis* TL01 plasmid pSHJGH1 right-hand end, NC_020894.1; 7, *S. hygroscopicus* subsp. *jinggangensis* 5008 plasmid pSHJG1 right-hand end, NC_017766.1; 8, *S. rimosus* ATCC 10970 chromosome, CP048261.1; 9, *S. rimosus* ATCC 10970 plasmid SRP1 left-hand end, CP048261.2; 10, *S. rimosus* ATCC 10970 plasmid SRP1 right-hand end, CP048261.2; 11, *Streptomyces pratensis* ATCC 33331 plasmid pSFLAO1, CP002476.1; 12, *S. griseus* 2247 chromosome; 13, *Streptomyces* sp. 769 plasmid pSGZL, CP003988.1. (**b**) The terminal 150 bp of 3′ replicon ends of Sg2247-type telomeres showing stem structures and hairpins identified using Mfold [28] are displayed. Revised free energies (ΔG, kJ mol^−1^) were determined using Jacobson–Stockmeyer theory to assign free energies to multi-branch loops. Those telomeres located on chromosomal (X) and plasmid (P) replicons are designated in parentheses. Further details of known Sg2247 telomeres are displayed in Table S2 and each individual Mfold representation in Fig S3.

To close the genome sequence of *S. rimosus* ATCC 10970, we employed a self-ligation PCR-sequencing method [23] to recover the telomeres of the chromosome and SRP1. Assembly of Illumina and PacBio reads produced two contigs corresponding to these replicons (see the Supplementary Information) and allowed us to identify SmaI and PvuII as restriction enzymes that digested close to the ends of both molecules. When the *S. rimosus* genomic DNA was digested with SmaI and PvuII and the fragments were self-ligated, circular molecules were generated that corresponded to the ends of both replicons (Fig. S2). Using the primers listed in Table S1, the telomeres were recovered as PCR products and sequenced. These telomere sequences were combined with the draft sequence of both replicons to close the sequences. Here, we define a closed streptomycete sequence to include the entire sequence of all replicons; many sequences listed as complete by the NCBI do not contain recognizable telomeres. The chromosomal telomeres had identical sequences, whilst the left- and right-hand telomeres of SRP1 had a high degree of similarity (Fig. 2) to each other and to a group of telomeres first found in *S. griseus* 2247 [11] that are distinct from archetypal and other non-archetypal telomeres. Although the telomeric sequence from this strain has not been submitted to the NCBI, we named this class of telomeres as Sg2247. An archetypal telomeric sequence exists in the NCBI database from the telomere of *S. rimosus* R6 [8] (accession number AY043328.1) and suggests that telomeric heterogeneity exists within strains of this species. *S. rimosus* ATCC 10970 and *S. rimosus* R6 are independent isolates: the former is the original soil isolate [39] and the latter is a soil isolate from Zagreb, Croatia [16].

We next set out to determine the incidence of Sg2247-type telomeres across the streptomycetes. We used the terminal 150 bp of the *S. rimosus* chromosome telomere to search the NCBI database using blastn. This generated over 50 hits, with a query coverage and identity of at least 19 and 76%, respectively. To exclude pseudo-telomeres [15, 40] and detect true telomeric sequences, we only included sequences that were found at the end of a replicon from complete genomes or where the telomere sequence had been physically isolated and sequenced. Eleven replicons contained telomeres similar to those of *S. rimosus* (Sg2247-type); two were at both chromosome ends (* S. rimosus* ATCC 10970 and *Streptomyces albidoflavus* J1074) [41], whilst ' *Streptomyces cattleya* ' NRRL8057 [42] possesses a Sg2247 telomere at one end and no recognizable telomere at the other. In addition, Sg2247-type telomeres were found at both termini of three plasmids and at one terminus of a further four plasmids. Interestingly, two plasmids (pSHJG1 from *Streptomyces hygroscopicus* subsp. *jinggangensis* 5008 [43] and pSHJGH1 from *S. hygroscopicus* subsp. *jinggangensis* TL01) contained a Sg2247-type telomere at one terminus and an archetypal telomere at the other.

A ClustalW alignment of 13 Sg2247 telomeres was used to determine the evolutionary relationships within this class of telomeres (Fig. 2a). Sg2247-type telomeres form similar stem-loop structures to those found in archetypal and non-archetypal telomeres [15], with up to eight stem-loop structures predicted within the terminal 150 bp and highest similarity at their 3′ ends where terminal stem-loop structures are highly conserved (Fig. 2, individual Mfold projections are shown in Fig. S3). The most common sequences found at the hairpins were GGA, GAA and GTA, like that found at the hairpins of archetypal telomeres [15]. Revised free energies (ΔG) of the stem-loop structures predicted by Mfold [28] varied between −36.51 and −58.46 kJ mol^−1^. Sg2247 telomeres fell into two groups that reflected their phylogenetic relationship (Fig. 2b): those with a relatively low ΔG (strains 1–7) and those with a significantly higher ΔG [strains 8–13, according to a Student's *t*-test (*P*<6.47×10^−7^)]. Strains with the lowest ΔG (strains 1–7) contained longer hairpins distal to the 3′ replicon end, although it is unclear whether this has any functional significance.

### Six classes of telomeres are found in the linear replicons of streptomycetes

Archetypal telomeres are found in many streptomycete replicons, such as the chromosome end of *S. coelicolor* M145 [25]. Non-archetypal telomeres were first identified in SCP1, the GLP of *S. coelicolor* A3(2) [15], and another class of telomeres was identified in the *S. griseus* 13350 chromosome [13], here termed Sg13350-type. Finally, two types of telomeres were found at the ends of two linear plasmids, pRL1 and pRL2 from streptomycete soil isolates [12]. Using the same approach with which we identified Sg2247-type telomeres, we also compiled a list of telomeres from all six classes that were found at the end of closed replicons or had been recovered physically and sequenced by Sanger sequencing [23]. The predicted streptomycete telomeres of all six classes are listed in Table S2. Archetypal telomeres are the most frequent and are found in both chromosomes and linear plasmids, whilst three SCP1-type telomeres were identified (SCP1) [15], pSCO2 [7] and pFRL3 (Chen and others, unpublished data, 2013; accession number, KF602048.1), all from linear plasmids. The chromosomal telomere of *S. griseus* 13350 [13] remains the sole member of the Sg13350 class, as do the telomeres of pRL1 and pRL2 of their eponymous classes [12].

Folding analysis of the six classes of telomeres indicated that all could form plausible stem-loop structures (Fig. 1 and individual Mfold projections in Fig. S4). Although there is little sequence similarity in the stems, the hairpins of the stem-loop structures across all six classes of telomeres are usually 3 or 4 nt in length, with GAA, GGA and GTA the most common hairpin. With the exception of Tap/Tpg [10] and Tac/Tpc [15], which prime end-patching of archetypal and SCP1-type telomeres, respectively, no other telomeric proteins have been characterized. Although a putative Tap was described in *S. albidoflavus* J1074 [41], we were unable to detect any similarity to Tap, Tpg, Tac or Tpc in genome sequences with Sg2247-type telomeres. This was also true in genomes with the Sg13350 and pLR1 types of telomeres, although pRL2 [12] encodes two candidate telomeric proteins: one displaying similarity to Tap and a helicase of *Thiobacillus* sp., whilst the other protein resembles Tpg and part of the adenovirus telomere terminal protein [12]. Taken together, this indicates that although streptomycete telomeres of all types bear some resemblance to each other in terms of the capacity of their 3′ overhangs to fold back on themselves, there are likely to be undiscovered systems able to catalyse end-patching.

We were only able to identify relatively few complete streptomycete genome sequences where all linear replicons were flanked by a member of these six telomere classes. Indeed, based on our strict criteria (complete sequence of all replicons and all replicons flanked by known telomeres), we could only confirm 20 closed genome sequences from streptomycetes. Many complete genomes within the NCBI database do not encode Tap and Tpg, so it is possible that other classes of telomeres await discovery. In a small-scale study using PCR, 8 out of 17 newly detected streptomycete linear plasmids lacked typical *tap* and *tpg* sequences and archetypal telomeres [12], but contained two undiscovered telomere types (pRL1-type and pRL2-type; Fig. 1). This suggests that novel streptomycete telomeres are common. If our analysis of replicon ends is representative of those of the entire family, it suggests that archetypal telomeres are the most common and phylogenetically widespread as they are found in two genera (* Streptomyces* and *Kitasatospora *) of the family *Streptomycetaceae *. In addition, it seems that Sg2247-types are less abundant and form a minority of the ends of both chromosomes and plasmids, whilst the other classes of telomeres are likely to be rare.

### Streptomycete telomeres are not distributed according to the evolutionary relatedness of different strains

**Fig. 3.**
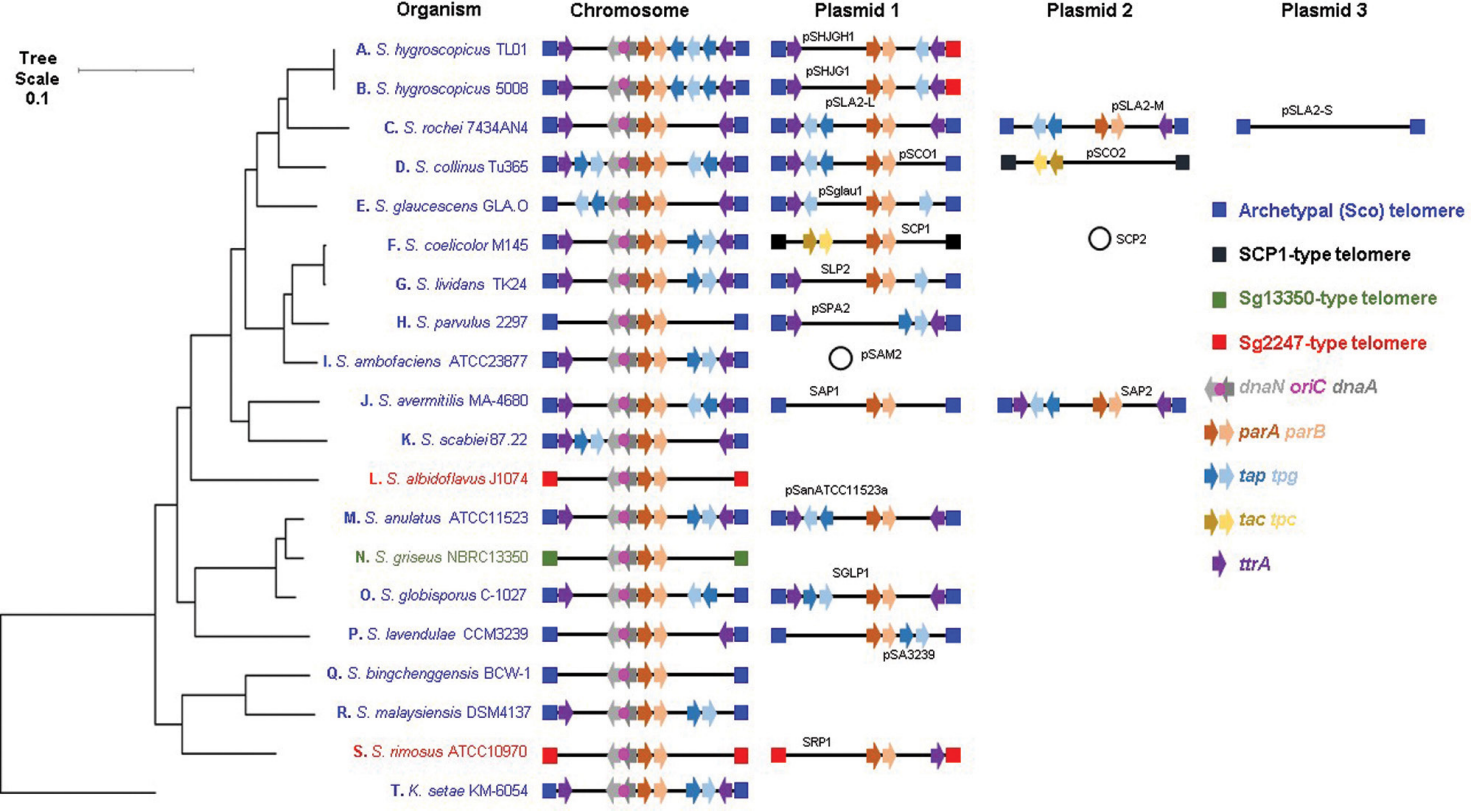
Relative positions of genes encoding telomeric and partitioning proteins with respect to *oriC* and telomere class in the family *Streptomycetaceae *. All replicons carried by the 20 organisms with closed genomes (both chromosomes and plasmids) were investigated for the presence of genes encoding the archetypal and non-archetypal telomeric proteins, Tap/Tpg and Tac/Tpc, respectively. In addition, genes encoding TtrA, DnaN, DnaA, ParA and ParB are plotted (Table S3). Four of the six different classes of streptomycete telomeres are also displayed (archetypal (Sco), blue; SCP1, black; Sg2247, red; Sg13350, green). The locations of these genes and telomeres were plotted in relation to the whole genome MLSA described in Fig. S5. Branch lengths represent the evolutionary time between two nodes (units: substitutions per sequence site).

To determine whether the six different classes of streptomycete telomeres were associated with different phylogenetic clades within the family *Streptomycetaceae *, we first compiled a list of closed streptomycete sequences. As defined above, a closed genome sequence was that all replicons of that organism must be completely sequenced and that each linear replicon was flanked at both ends by one of the six classes of telomeres described previously. At the time of analysis (April 2020), there were 223 streptomycete genomes that were listed as complete in the NCBI database (1 from the genus *Streptacidiphilus *, 4 from *Kitasatospora* and 218 from *Streptomyces *). After inspecting these genomes for the presence of telomeres located at both ends of all linear replicons, we identified 20 strains that met these criteria (Table S3). The replicons of other genome sequences recorded as complete in the NCBI database might be flanked by undiscovered telomeres. As the 5′ ends of streptomycete replicons are covalently bound to a telomeric protein, sequencing approaches, such as Illumina, that employ paired-end reads may struggle to sequence to the very ends of linear replicons. In addition, the presence of TIRs flanking linear replicons may create assembly challenges using software tools designed for circular replicons. Taken together, it is unsurprising that many streptomycete genome sequences are not closed. We next organized all linear replicons carried by these 20 strains so that they were placed in the same direction based on the *oriC* region of *S. coelicolor* [25, 44] (Fig. 3). Replicons were orientated so that *dnaN* and *dnaA* were transcribed with the bottom strand as the coding strand, and *parA* and *parB* transcribed with the top strand as the coding strand. Chromosomes were organized in this orientation to comply with the established orientation for *S. coelicolor* M145, which has *SCO0001* on the left and *SCO7846* on the right [25]. Subsequently, we organized the 20 closed strains phylogenetically using OrthoANI [30] and AutoMLST [29] with *K. setae* KM-6054 as an outgroup by generating a maximum-likelihood tree and determining the location of strains with different classes of telomeres on this tree (Fig. S5). The high-resolution species tree shows that although archetypal telomeres are the most common of the chromosome ends, two phylogenetically separated strains (* S. albidoflavus* J1074 and *S. rimosus* ATCC 10970) carry chromosomes with Sg2247-type telomeres and suggests that telomeric exchange through horizontal gene transfer has occurred. The telomeres of *S. griseus* NBRC 13350 were confirmed by physical recovery [13] and, at the time of writing, no other telomeres with similarity to the ends of this strain have been identified. Despite this, the closely related strains *Streptomyces anulatus* ATCC 11523 and *Streptomyces globisporus* C-1027 (Fig. S5) possess archetypal telomeres that also indicates that horizontal transfer of telomeres has taken place. Similarly, *S. rimosus* ATCC 10970 (Sg2247), *Streptomyces malaysiensis* DSM4137 (archetypal) and '*Streptomyces bingchenggensis*' BCW-1 (archetypal) are also phylogenetically related but possess telomeres from different classes. The only closed streptomycete genome from outside the genus *Streptomyces* is from *K. setae* KM-6054 with archetypal telomeres; there are no characterized telomeric sequences available for *Streptacidiphilus *. Both *S. coelicolor* M145 and *Streptomyces collinus* Tu 365 possess chromosomes with archetypal telomeres, but plasmids with SCP1-type telomeres (Fig. 3). Intriguingly, the two closely related strains of *S. hygroscopicus* subsp. *jinggangensis* [43] possess a chromosome with archetypal telomeres, but a hybrid GLP with one archetypal and one Sg2247-type telomere. Hybrid replicons are not unknown in streptomycetes as demonstrated by the construction of *S. coelicolor* 2106 that carries a 1.85 Mb GLP (derived from SCP1), in addition to a 7.2 Mb linear hybrid chromosome with one archetypal end from the chromosome and one SCP1-type end from the GLP and, thus, this strain possesses replicons with both archetypal and non-archetypal telomeres [45].

We used this collection of closed genomes to investigate the TIRs by alignment of both ends of all replicons. The 20 chromosomes had a mean size of 8 846 536.25 bp (range 6 841 649–11 936 683 bp) and were flanked with 37 990 (range 13–237 155 bp) perfect inverted repeats and 77 092 (range 138–631 365 bp) imperfect inverted repeats, whilst the 16 GLPs spread over 12 strains were on average 188 460 bp in size (range 17 526–617 085 bp) with TIRs with 5775 bp mean perfect inverted repeats and 6552 bp mean imperfect inverted repeats. On average, a perfect TIR constituted 0.34% (0.87% imperfect) of a streptomycete chromosome and 3.06% (3.48% imperfect) of a GLP.

Whilst all chromosomes encode *dnaN*, *dnaA*, *parA* and *parB*, not all plasmids carry *parAB* (pSLA2-S, *Streptomyces rochei *; pSCO2, *S. collinus *; and pSPA2, *Streptomyces parvulus *); presumably these plasmids do not require these proteins for partitioning, or their functions are provided *in trans* by the chromosomal copies of these genes. We also mapped genes encoding known telomeric proteins (*tap*/*tpg*, *tac*/*tpc* and *ttrA*) (Fig. 3). SCP1-type telomeres flank two replicons, the GLPs SCP1 from *S. coelicolor* and pSSCO2 from *S. collinus *. Neither of these replicons encode Tap, Tpg nor TtrA, whilst both carry *tac* and *tpc*, encoding known non-archetypal telomeric proteins [15]. Consistent with previous analysis of *S. albidoflavus* J1074 [41], we were unable to locate either *tap* or *tpg* in the genomes of the two strains with Sg2247-type telomeres (* S. albidoflavus* J1074 or *S. rimosus* ATCC 10970) or *S. griseus* 13350 [13]. This suggests that their end-patching is carried out by a different mechanism to that of archetypal or SCP1-type telomeres and the identity of the proteins that catalyse this process remains elusive at the present time. The two closely related strains of *S. hygroscopicus* subsp. *jinggangensis* [43] contain plasmids with one archetypal end and one Sg2247-type telomere. These two strains carry two copies of *tap* on the chromosome and a copy of *tpg* on both the chromosome and plasmid. All strains with archetypal telomeres encode Tap and Tpg on at least one replicon, with the exception of '*S. bingchenggensis*' BCW-1 [46]; it is unclear how this strain primes replication at the chromosome ends. Some strains (* S. rochei* 7434AN4 [47, 48], *S. parvulus* 2297, *Streptomyces lavendulae* CCM3239) do not carry *tap* and *tpg* on the chromosome, but do so on at least one GLP, suggesting that these plasmids are required to encode the telomeric proteins for chromosomal end-patching. Conversely, several GLPs do not encode telomeric proteins: SLP2 from '*Streptomyces lividans*' (Tap), pSglau1 from *Streptomyces glaucescens* GLA.0 (Tap), pSLA2-S from *S. rochei* 7434AN4 (Tap and Tpg) and SAP1 from *Streptomyces avermitilis* (Tap and Tpg), and suggest that end-patching is carried out by proteins encoded *in trans* or by other unknown proteins. Nevertheless, it seems that there is some crosstalk of the telomeric proteins and telomeres of different replicons contained within an individual strain.

Most replicons are flanked by inwardly transcribed copies of *ttrA*, a putative DEAD-box helicase that is required for conjugation of the GLP, SLP2 in '*S. lividans*' [40]. When present, *ttrA* is often found towards the ends of replicons that are flanked by archetypal telomeres and is transcribed in an inward direction, suggesting that its orientation with respect to the telomeres is important for its function or transcriptional regulation. Exceptions to this are the *S. parvulus* 2297 and '*S. bingchenggensis*' BCW-1 chromosomes, although the former strain carries a plasmid (pSPA2) that perhaps provides TtrA functionality *in trans*. This gene is less common at the ends of replicons with non-archetypal, Sg13350- or the Sg2247-type replicon of *S. albidoflavus* J1074. Despite this, S. *rimosus* encodes a copy of *ttrA* at each chromosome termini and one copy at the right end of SRP1; unusually, the latter gene is transcribed in an outward direction. Consequently, the role of TtrA in plasmid-mediated conjugation and presence of *ttrA* at both ends of most streptomycete replicons suggests that telomere exchange may have arisen through recombination of recipient chromosomes with incoming GLPs. The circular genetic maps of streptomycetes provides circumstantial evidence for association between chromosome ends and is supported by fluorescence *in situ* hybridization studies in *S. coelicolor* [49]. The telomeric proteins display both an intramolecular (Tpg–Tpg) and an intermolecular (Tpg–Tpc) physical association between different telomeric proteins that was demonstrated by chemical cross-linking [46]. This presents a problem for replicon segregation through the formation of replicon pseudo-dimers brought about by the *in trans* association of the telomeric proteins. In a mechanism first proposed in 2011, these pseudo-dimers can only be resolved through disassociation and association of the telomeric proteins with those of the other daughter replicon or the occurrence of recombination between the two replicons in order to resolve the pseudo-dimer [46]. The appearance of replicons with different telomeres reported here (Fig. 3) might then be brought about by resolution of pseudo-dimers by recombination. Of course, exchange of different telomeres might also occur by classical transposition or recombination alone [50], but it is difficult to see how pseudo-dimers might be resolved without telomeric protein exchange or recombination between the two replicons [46].

Genome plasticity and evolution of streptomycetes is generated through intrinsic spontaneous recombination in addition to horizontal gene transfer in the environment and actioned by non-homologous end joining and integration of genetic material at the healing site [51]. Following a recent investigation of natural streptomycetes, it was revealed that closely related strains showed a large diversity of TIRs that reflected telomere exchanges between the chromosome and GLPs, and suggests that streptomycete telomeres display extensive allelic exchange brought about by horizontal gene transfer [52]. Strains that have undergone chromosomal changes produce diversified secondary metabolites and secrete more antibiotics [53], so perhaps it is the capacity for genetic exchange brought about by linear replicons that contributes to the metabolic diversity and bioactivity of this bacterial group.

Consequently, our analysis of closed streptomycete genome sequences demonstrates telomeric incongruence with a whole-genome-based phylogeny of these strains. This fact, taken together with the consistency of the location of *ttrA* at both chromosomal and plasmids ends, coupled with the ability of GLPs to form hybrid ends (* S. hygroscopicus* subsp. *jinggangensis*), suggest that telomeric exchange between streptomycete replicons is a common occurrence.

### A conserved origin island lies at the centre of streptomycete chromosomes

When the 20 closed streptomycete chromosomes were orientated with *dnaA* and *dnaN* transcribed in a right to left direction, we noticed that *parA* and *parB* were transcribed in the opposite direction (Fig. 3). This suggested that the region containing *oriC* possessed conserved synteny across the analysed sequences. For this reason, we decided to investigate the extent of this synteny by carrying out a progressive alignment of the 20 closed chromosomes using Mauve [31]. This analysis showed a ~50 kb locally collinear block (LCB), containing *oriC*, that lay in the core region of all chromosomes (brown arrow, Fig. S6), whilst other LCBs (green and purple arrows, Fig. S6) in the flanking regions displayed bilateral symmetry around *oriC*. For example, the green and purple LCBs occur in a similar orientation flanking *oriC* in closely related strains. In general, the green and purple LCBs are located on opposite replichores, except for the related strains '*S. bingchenggensis*' BCW-1 and *S. malaysiensis* DSM4137 where both the green and purple LCBs are located on the same replichore. This suggests that recombination cannot occur within this core (brown) LCB and that, at the core of streptomycete chromosomes, there is a conserved region of ~50 kb that we have designated the origin island.

**Fig. 4.**
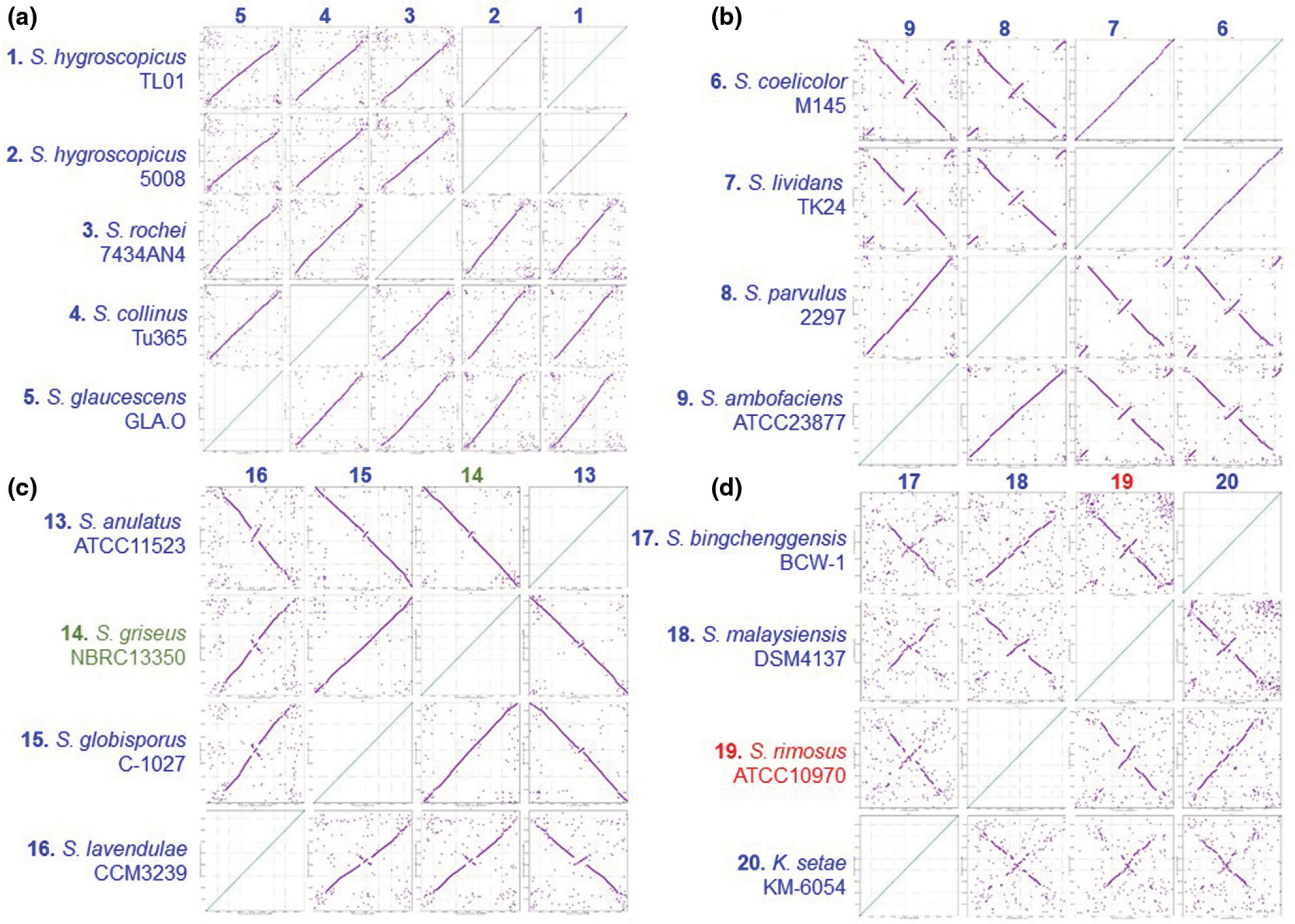
Related strains display both syntenous and asynetenous chromosomes. Each of the 20 closed streptomycete chromosomes, organized so that the *oriC* regions were syntenous, were submitted to Nucmer and dot plots generated [32]. A comparison of all sequences is displayed in Fig. S7. Dot plots of strains around selected phylogenetic nodes (Fig. S5) are displayed here. (**a**) 1, *S. hygroscopicus* subsp. *jinggangensis* TL01; 2, *S. hygroscopicus* subsp. *jinggangensis* 5008; 3, *S. rochei* 7434AN4; 4, *S. collinus* Tu 365; 5, *S. glaucescens* GLA.O. (**b**) 6, *S. coelicolor* M145; 7, '*S. lividans*' TK24; 8, *S. parvulus* 2297; 9, *S. ambofaciens* ATCC 23877. (**c**) 13, *S. anulatus* ATCC 11523; 14, *S. griseus* subsp. *griseus* NBRC 13350; 15, *S. globisporus* C-1027; 16, *S. lavendulae* subsp. *lavendulae* CCM3239. (**d**) 17, '*S. bingchenggensis*' BCW-1; 18, *S. malaysiensis* DSM4137; 19, *S. rimosus* ATCC 10970; 20, *K. setae* KM-6054.

We next investigated chromosome synteny in more detail through the generation of dot plots using Nucmer [32], where the chromosomal synteny of all strains was compared with each other and organized according to the MLSA tree for the 20 closed chromosomes displayed in Fig. S5. Dot plots for all 20 chromosomes are shown in Fig. S7 and for selected phylogenetic nodes in Fig. 4 that best illustrated chromosome synteny between related strains. Comparison of dot plots between sequences from related strains showed synteny across the entire length of the chromosomes, such as with the *S. hygroscopicus* node (Fig. 4a). Synteny is reduced towards the telomeres in comparison with the core region of the chromosome and likely reflects the genetic compartmentalization of streptomycete chromosomes that is consistent with the demonstration of a link between chromosome folding and gene expression in *Streptomyces ambofaciens* [54]. However, comparison of strains located at other phylogenetic nodes (Fig. 4b–d) shows that one or more major chromosome inversions had taken place during the evolutionary divergence of *S. coelicolor */'*S. lividans*' and *S. parvulus */* S. ambofaciens* (Fig. 4b). Similar inversions were also identified at the *S. griseus* (Fig. 4c) and *S. rimosus* nodes (Fig. 4d). Interestingly, although the *S. griseus* 13350 chromosome carries the unique Sg13350 telomere class, its high average nucleotide identity with both *S. anulatus* and *S. globisporus *, which both possess archetypal telomeres (Fig. S5), suggests that telomere exchange was a relatively recent evolutionary event. The obvious bilateral symmetry between related chromosomes suggests that the recombination events leading to these rearrangements take place between the two replichores on either side of the origin island. It may be, therefore, that the conserved synteny of the origin island represents an irreducible axis around which recombination can take place, whilst not being subject to recombination itself. It also suggests that maintenance of an intact origin island is necessary for successful chromosome replication, segregation or appropriate compaction [54].

**Fig. 5.**
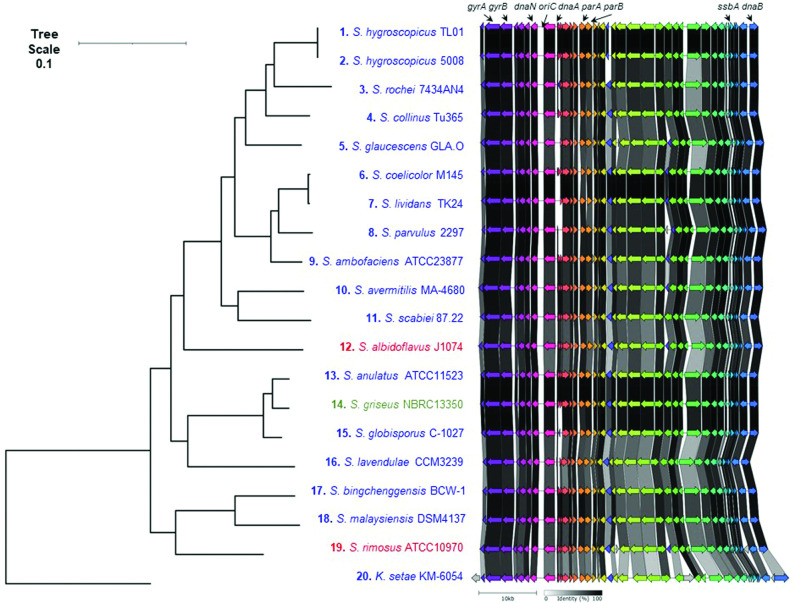
Streptomycete origin islands are syntenous. Origin islands from tRNA-ile (left) and *dnaB* (right, *SCO3911*) of 20 closed streptomycete genome sequences. Chromosomes with archetypal telomeres are listed in blue, Sg2247 telomeres in red and Sg13350 in green. Branch lengths represent the evolutionary time between two nodes (units: substitutions per sequence site).

In *S. coelicolor *, this origin island contains the region corresponding to *SCO3872–SCO3911* (Fig. 5) and includes *gyrA*, *gyrB*, *recF*, *dnaN*, *oriC*, *dnaA*, *parA*, *parB*, *ssbA and dnaB*. Across all 20 strains, the mean origin island size (Table S4) was 51 055 bp (range 48 019–57 671 bp) and constituted 0.59% of the chromosome (range 0.44–0.66%), and its location was on average 48.49% (range 41.25–53.25%) from the left-hand end of the replicon. Multiple genome alignments of streptomycete chromosomes show that they contain highly conserved core regions and variable sub-telomeric regions [55], and it has previously been reported that the central parts of streptomycete chromosomes are highly syntenic [56]. Whilst symmetrical rearrangements of streptomycete chromosomes have been reported before [41, 43], this is the first time, to our knowledge, that they have been related to a central axis. This suggests that the integrity of the origin island is important for maintenance of correct chromosome structure and function as when chromosome inversions take place, they do so in a symmetrical manner (between replichores) rather than in an asymmetrical manner (within a replichore).

Bacterial chromosomes display a high degree of organization with respect to the locations of core genes [54, 57]. In *Bordetella pertussis *, with a circular chromosome, there is a bias toward symmetrical inversions [58] for the maintenance of replichore balance and preservation of favourable gene arrangements. Core-gene-defined genome organizational frameworks (cGOFs) can be classified as either symmetric or asymmetric with respect to their orientation in relation to the origin-terminus axis [59]; in Gram-positive organisms, cGOFs are exclusively symmetric and reversible in orientation [59]. As a result, it seems that the bilateral symmetry of streptomycete chromosomal rearrangements is consistent with Gram-positive bacteria with circular chromosomes and that a bias exists towards maintaining an approximate equality in the lengths of both replichores.

### Asymmetric distribution of *parS* sites in streptomycete chromosomes

The delineation of the streptomycete origin island (Fig. 5) showed that this ~50 kb region not only contained *oriC* between *dnaN* and *dnaA*, but also a high number of putative *parS* sites located to the right of *oriC*. ParB binds to these sites and facilitates chromosome partitioning and connection with the polarisome [20, 21]. As a result, we analysed the location of *parS* sites across the 20 closed streptomycete chromosomes. To do this, we employed a consensus matrix developed to locate putative prokaryotic *parS* sites [34].

**Fig. 6.**
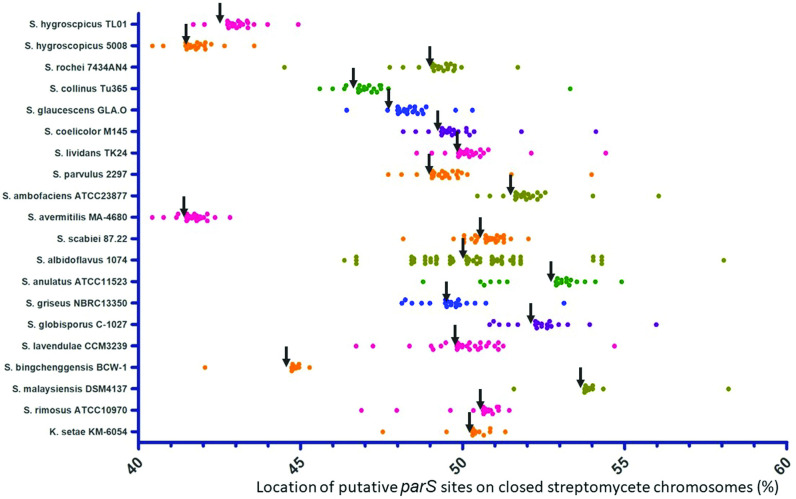
Location of *parS* sites in the central 20% of streptomycete chromosomes. The locations of predicted *parS* sites on the chromosomes of the 20 closed streptomycete genomes were determined by interrogation of those genomes with the consensus matrix for bacterial *parS* sites [34]. Locations were expressed as a percentage of the chromosome size. The last base of *dnaA* from all genomes was selected as the location of *oriC* (black arrows).

The locations of these sites within the core region of the chromosome and with respect to *oriC* are shown in Fig. 6. The locations and sequences of these putative sites across all 20 closed genomes is provided in Supplementary File S1. On average, the number of *parS* sites per chromosome was 22.8 (range 11–65); most bacteria contain less than 5 *parS* sites [34], so the fact that streptomycetes carry up to 65 *parS* sites (* S. albidoflavus* J1074) [41] is remarkable. This strain, originally named *Streptomyces albus* J1074 [60], displays fast and dispersed growth in liquid culture [41]; it may be that the 65 predicted *parS* sites found in this strain contribute towards this through more efficient chromosome segregation and branching. As in *S. coelicolor* [20], most *parS* sites are located close to *oriC* in all strains and the origin island is abundant with *parS* sites; on average 45.24% of *parS* sites are located within the origin island with a mean distance of 5602 bp between *parS* sites in the origin island and 1 113 078 bp between *parS* sites in the bulk chromosome. Significantly, there is also a bias in the location of *parS* sites towards the right-hand side of the chromosome (Fig. 6). A total of 20.14% of *parS* sites lie to the left of *oriC* and 79.86% to the right (Table S4), meaning that during DNA replication and partitioning it is likely that the right replichore carries a greater abundance of ParB/*parS* nucleoprotein complexes with potential implications for chromosome segregation and connection of the chromosome to the hyphal tip. We did not include *Streptomyces venezuelae* in our analysis as the telomeres of its chromosome have not yet been established [61]; although, the possession of *tap* and *tpg* suggests that they resemble the archetypal telomeres of *S. coelicolor* M145. However, ChIP-seq analysis confirmed the formation of large nucleoprotein ParB complexes located at 16 *parS* sites in *S. venezuelae* 10712 [62] and, recently, the role of ParB in promoting chromosome inter-arm contact was reported [63]. *parS* sites are also located to the right of *oriC* in the *S. venezuelae* chromosome (data not shown). The significance of this bias of *parS* sites on the right replichore is unclear; it is true that only one daughter chromosome is directed towards the hyphal tip by ParB in *S. coelicolor* [21], but it is difficult to explain how a bias in ParB binding to the right replichore would manifest itself in the preferential direction of one daughter chromosome toward the hyphal tip.

### Conclusion

In summary, following the completion of the *S. rimosus* ATCC 10790 genome sequence, the parental strain of an important lineage of industrial bacteria, we first characterized an uncommon set of telomeres for this organism before comparing them to the other five classes of streptomycete replicon ends. Through the organization of the closed replicons of 20 streptomycete chromosomes with respect to their phylogeny and physical orientation, we determined that the telomeres were not associated with particular clades and were likely shared amongst different strains by horizontal gene transfer. We identified an origin island that forms an axis around which symmetrical chromosome inversions can take place and that there is a bias in *parS* sites to the right of *oriC* in closed streptomycete genomes. These results open up new questions for further investigations towards understanding the mechanisms of end-patching of streptomycete chromosomes and the biological meaning that lies in the *parS* site bias. As such, understanding the fundamental structure of genome organization and mechanisms will prove indispensable in strain engineering for improved specialized metabolite production by this industrially important microbial group.

## Supplementary Data

Supplementary material 1

Supplementary material 2
