# Supplementary material for: Bilateral symmetry of linear streptomycete chromosomes
Source: Microb Genom. 2021 Nov 15;7(11):000692. doi: 10.1099/mgen.0.000692 (PMC8743542; doi:10.1099/mgen.0.000692)
Supplement: Supplementary material 1 [file mgen-7-0692-s001.pdf]

<u>**Supplementary Information**</u>

Bilateral symmetry of linear streptomycete chromosomes

Lis Algora, Jana Schniete, David Mark, Iain Hunter, Paul Herron

Assembly of the *S. rimosus* ATCC 10970 closed genome sequence

The complete genome sequence of *S. rimosus* ATCC10970, was assembled using a combination of reads from long-read PacBio sequencing and short-read Illumina sequencing. In the first instance, assembly of PacBio data was carried out through HGAP using CCS reads generated from this raw data, with a minimum of 3 reads through a single molecule; this yielded 19555 sequences between 48 and 16693bp in length. Meanwhile Illumina sequencing generated 2,415,879 reads with a mean coverage of 101.793x.

PacBio sequences were assembled into five contigs using HGAP (Contig A, 3,195,161bp; Contig B, 2,836,700bp; Contig C, 2,083,116bp; Contig D, 1, 240,339bp and Contig E, 278,157bp). Contig E was identified as the known *S. rimosus* G7 Giant Linear Plasmid (GLP) due to its similarity in size to the known GLP of this strain; estimated to be slightly smaller than the 312Kb GLP of *S. rimosus* R6 determined by pulsed-field gel electrophoresis (PFGE) (1). In addition, this contig also contained copies of *parA*, *parB* and *traSA* characteristic of streptomycete GLPs (2)

Contigs A-D were joined using data from publically available data from two *S. rimosus* draft genome sequences (*S. rimosus* ATCC 10970, DDBJ/EMBL/GenBank GenBank accession number: ANSJ00000000 (3) and *S. rimosus* NRRL ISP5260, DDBJ/EMBL/GenBank: JNYR00000000.1). Contigs A and B were joined using the bridging contig NZ_ANSJ01000266.1 and Contigs B and C using the bridging contig NZ_ANSJ01000079.1|. The other end of Contig C carried an rRNA operon as did the telomere-distal end of Contig D, but we were unable to identify existing overlapping contigs from other sequencing projects. As a result, the hybrid assembler Unicycler was used to generate an assembly of both long PacBio and short Illumina sequences and generated 56 contigs; one of these contigs (Contig 2, 1,201,767bp) spanned Contigs C and D allowing assembly the chromosome as a single contig. Contig A and Contig D contained ~11Kb Terminal Inverted Repeat (TIRs) which suggested that these contigs represented the terminal contigs. This was confirmed by the identification of two contigs, NZ_JNYR01000065 and NZ_JNYR01000071, that overlapped with the TIRs of Contig A and Contig D respectively. This allowed us to further extend the sequence of the TIRs. The sequence of both of these two contigs were also found at the *oriC*-distal ends of both Contig C and Contig D. Contig E was extended through identification of the bridging contigs NZ_ANSJ01000106.1| and NZ_ANSJ01000002.1. Despite this, the ends of

the draft linear chromosome did not contain either archetypal or non-archetypal telomeres. As a result we independently recovered and sequenced the telomeres of both the GLP and chromosome (see materials and methods). Comparison of the telomeric sequences with our assembled contiguous chromosomal and plasmid contigs allowed us to extend the contiguous sequences to close the sequence of both replicons.

Following the construction of a draft sequence, the sequence was polished by first using Bowtie (Galaxy version 1.2.0) (4) where Illumina paired end reads (FastQ files were aligned with the draft sequence to generate a BAM files. This file was then mapped to the draft sequence files (both chromosome and plasmid) using Pilon in Galaxy Version 1.20.1,(5) to generate the final sequences of the two replicons. Annotation was carried out using NCBI Prokaryotic Genome Annotation Pipeline (PGAP) and is available as Bioproject PRJNA182749 and Biosample SAMN02471950. The *S. rimosus* ATCC 10970 chromosome is listed under accession number CP048261 and the plasmid, pSRP1, CP048262.

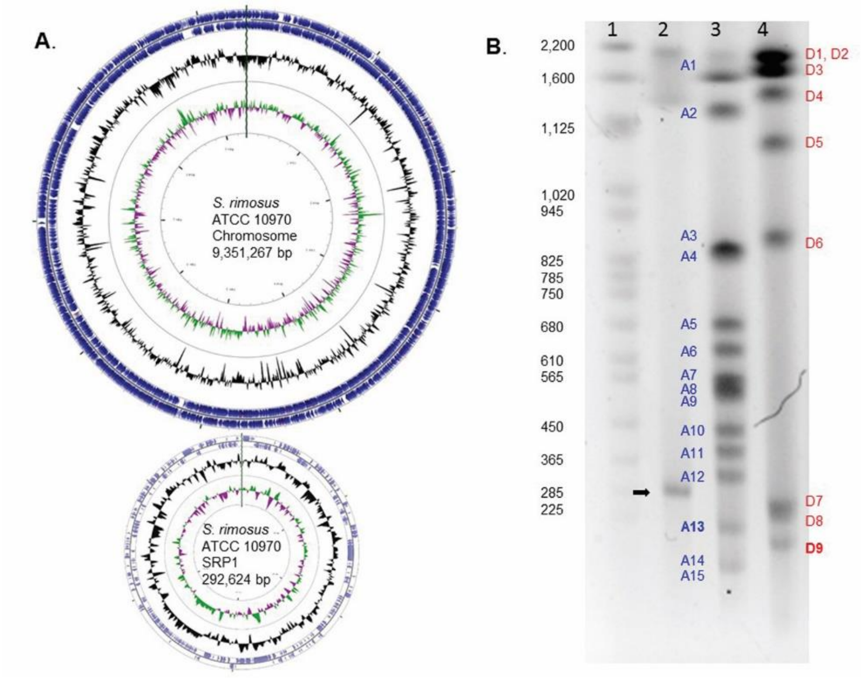

AseI Fragment	Size (bp)	DraI Fragment	Size (bp)
A1	2,268,128	D1	2,162,072
A2	1,136,413	D2	2,029,716
A3	831,533	D3	1,563,320
A4	827,548	D4	1,184,854
A5	682,802	D5	1,052,419
A6	623,988	D6	859,144
A7	551,863	D7	263,506
A8	527,971	D8	236,236
A9	513,659	**D9**	**172,852**
A10	433,169	**D10**	**43,122**
A11	382,749	**D11**	**30,090**
A12	320,709	**D12**	**25,966**
**A13**	**207,231**	**D13**	**19,753**
A14	127,494	**D14**	**841**
A15	123,241		
**A16**	**59,216**		
**A17**	**26,177**		

Fig S1. The closed genome sequences of the linear chromosome and giant linear plasmid, SRP1, from *S. rimosus* ATCC10970. (A) The linear replicons are represented as circles with the telomeres at 12 o'clock and *oriC* at 6 o'clock. From outside to inside, the concentric circles represent: nucleotide position on genome sequence; coding sequences (CDS) on the forward strand; CDS on the reverse strand; GC content (black); GC-skew on forward strand (purple); GC skew on the reverse strand (green). The genome sequence is listed in NCBI under accession number CP048261**. (B)** Pulsed Field Gel Electrophoresis analysis of *S. rimosus* ATCC10970. The size of SRP1 (lane 2, black arrow) was verified by Pulsed Field Gel Electrophoresis in comparison to yeast chromosomal markers (Biorad, lane 1). Restriction digestion with AseI (lane 3) and DraI (lane 4) of *S. rimosus* mycelial plugs followed by PFGE, allowed us to corroborate the genome assembly by comparison of the observed fragments with the *in silico* predictions of band sizes.

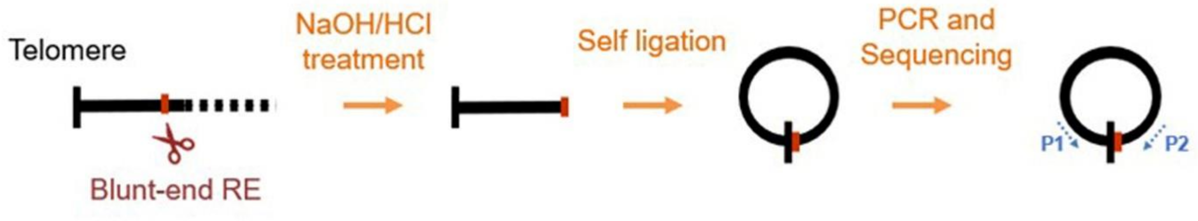

Fig S2. Scheme for recovery of *S. rimosus* telomeres. In order to complete the genome sequence of *S. rimosus* we employed self-ligation of blunt ends coupled with inverted PCR (primers P1 and P2) and sequencing of the resulting amplicons (6) to recover the telomeres of the chromosome and SRP1.

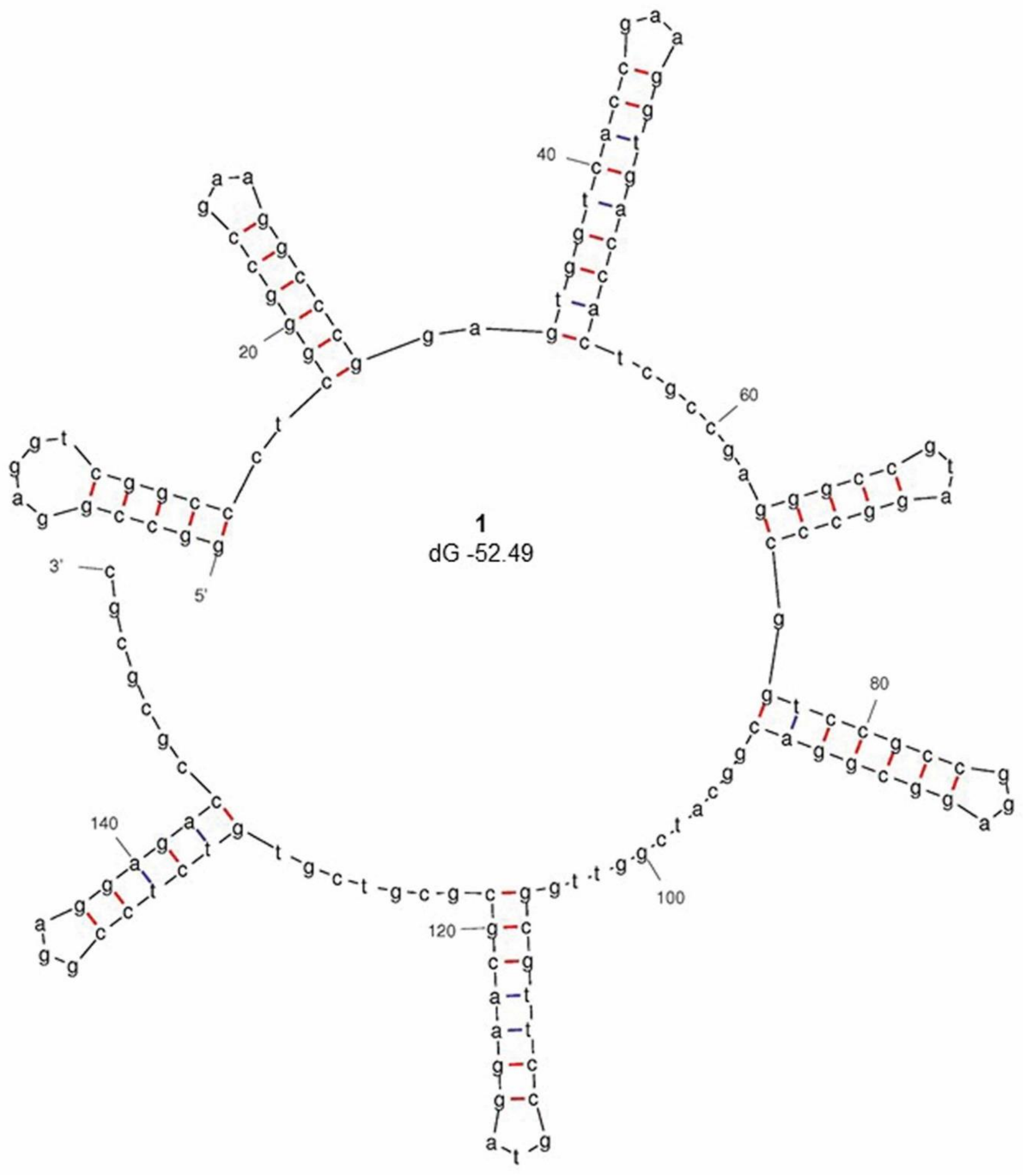
1
dG -52.49
5'
3'
20
40
60
80
100
120
140

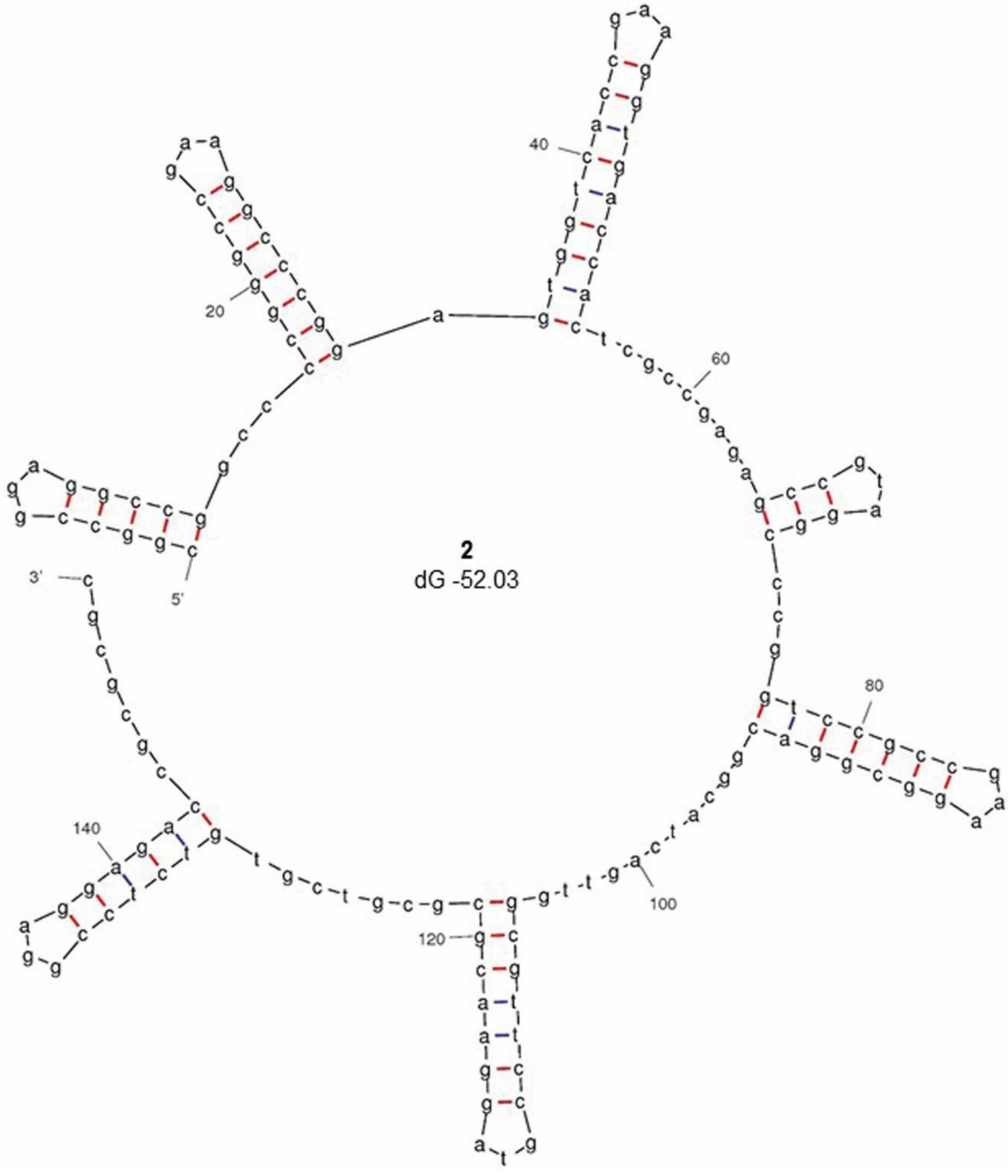

2
dG -52.03
5'
3'
20
40
60
80
100
120
140

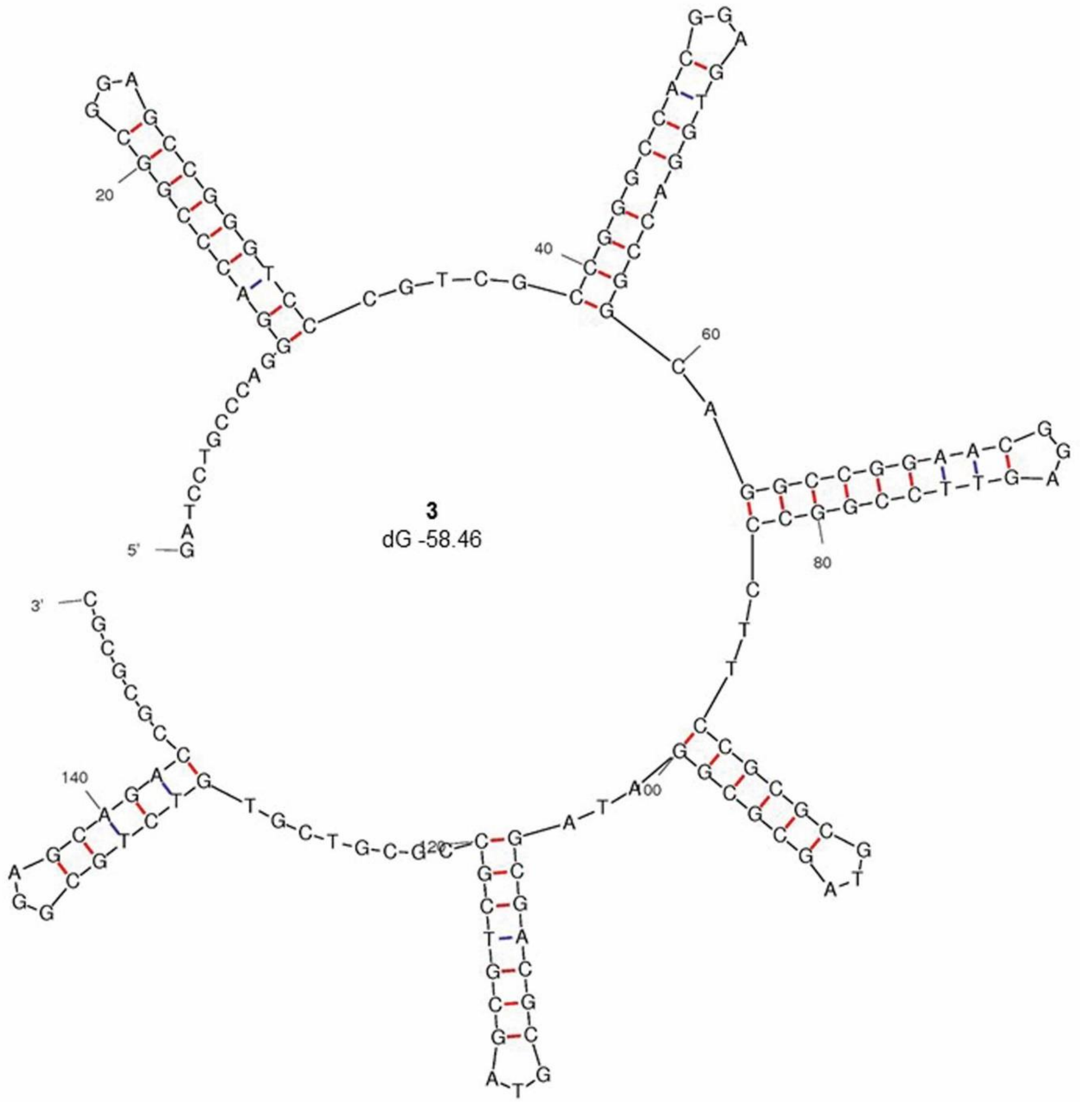
3
dG -58.46
5'
3'
20
40
60
80
100
120
140

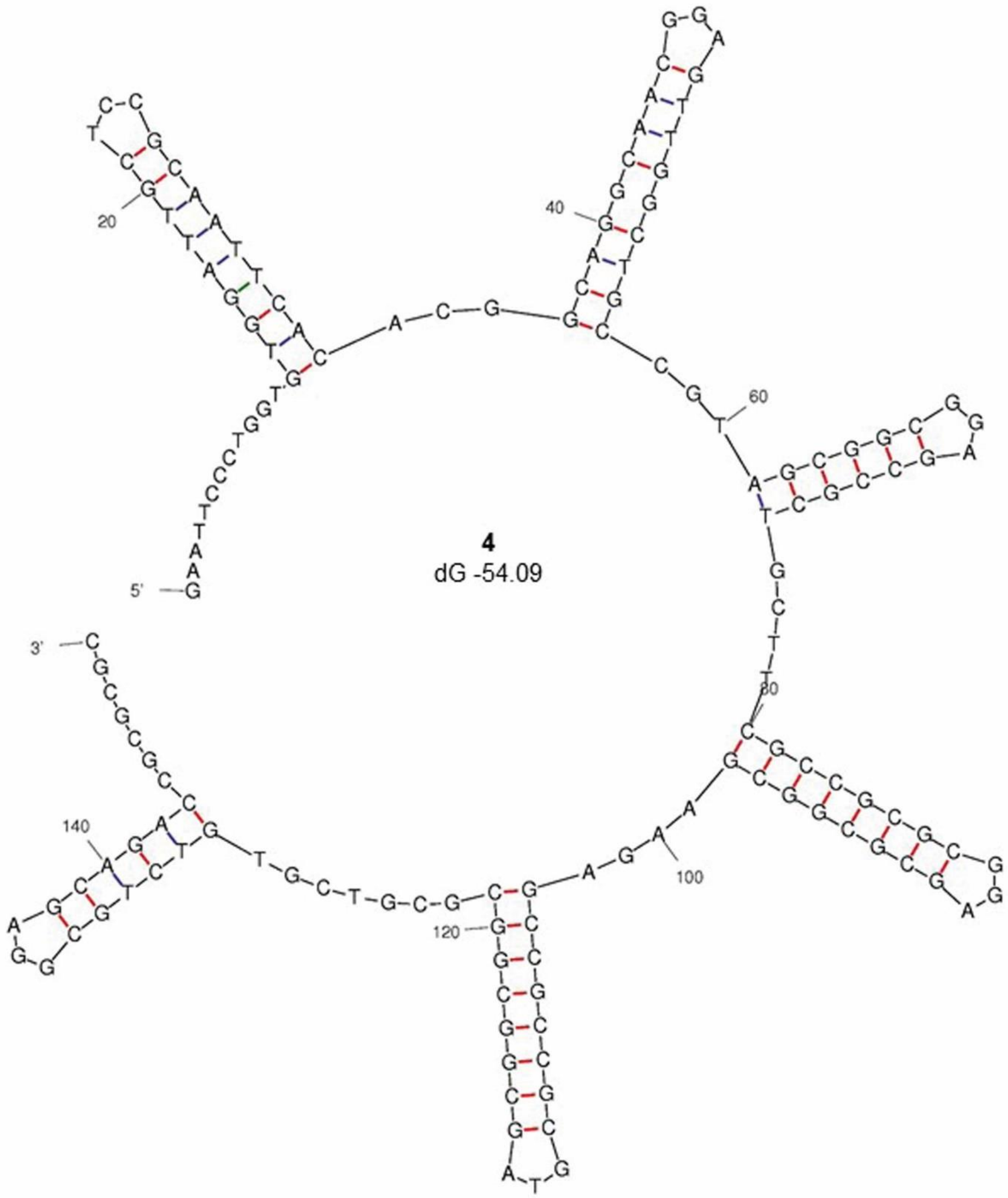
4
dG -54.09
5'
3'
20
40
60
80
100
120
140

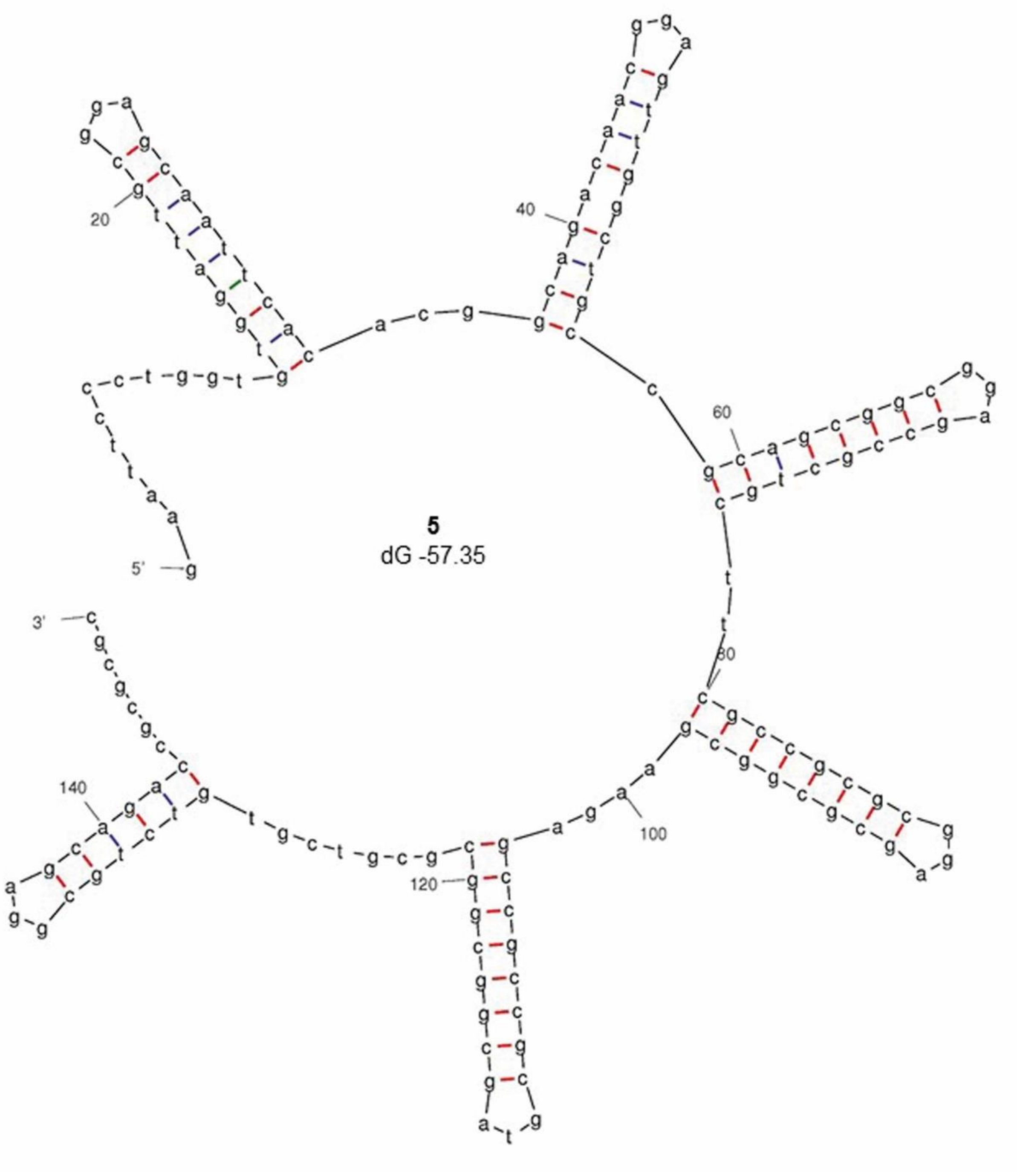
5
dG -57.35

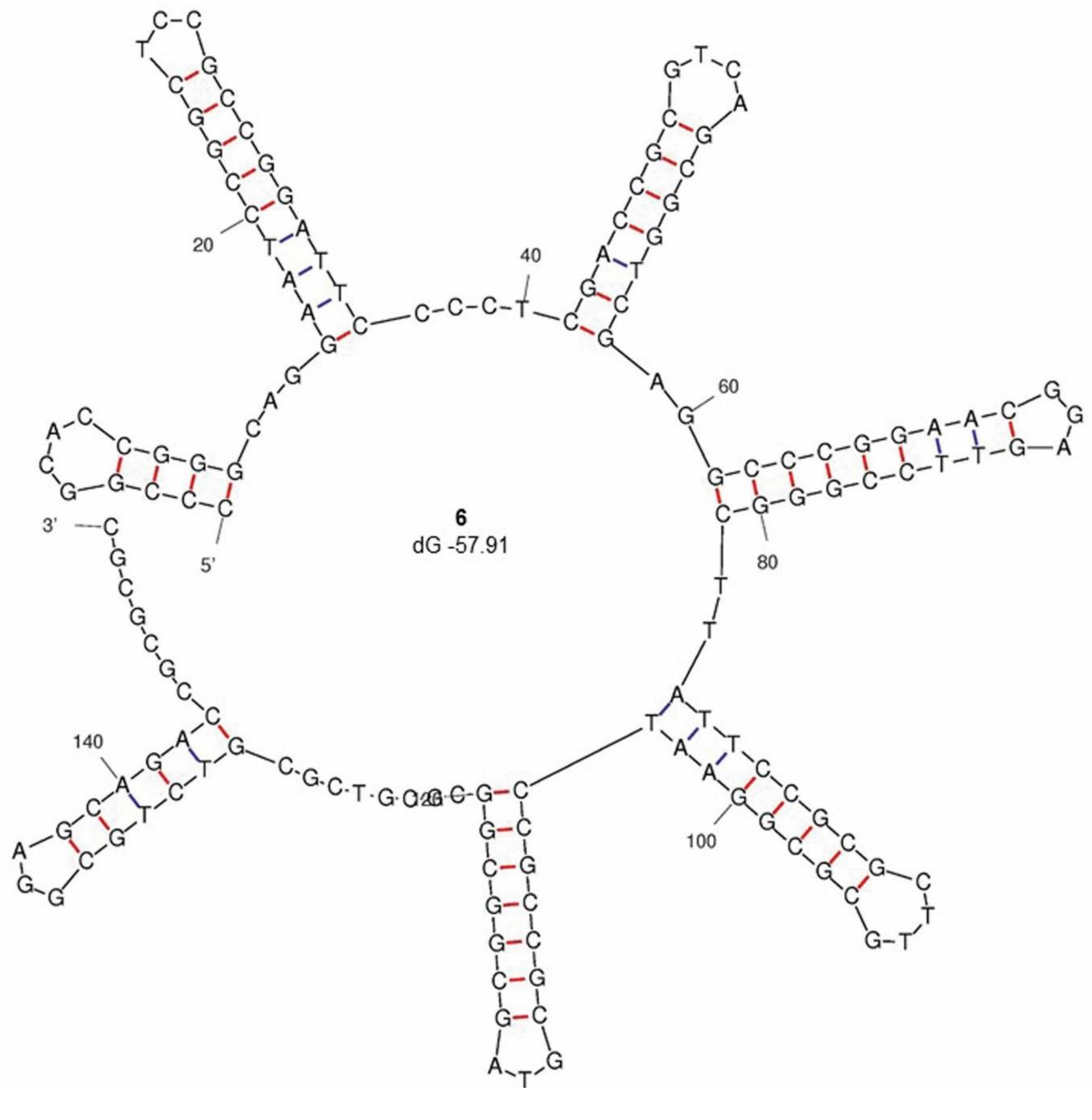
6
dG -57.91
5’
3’
20
40
60
80
100
140

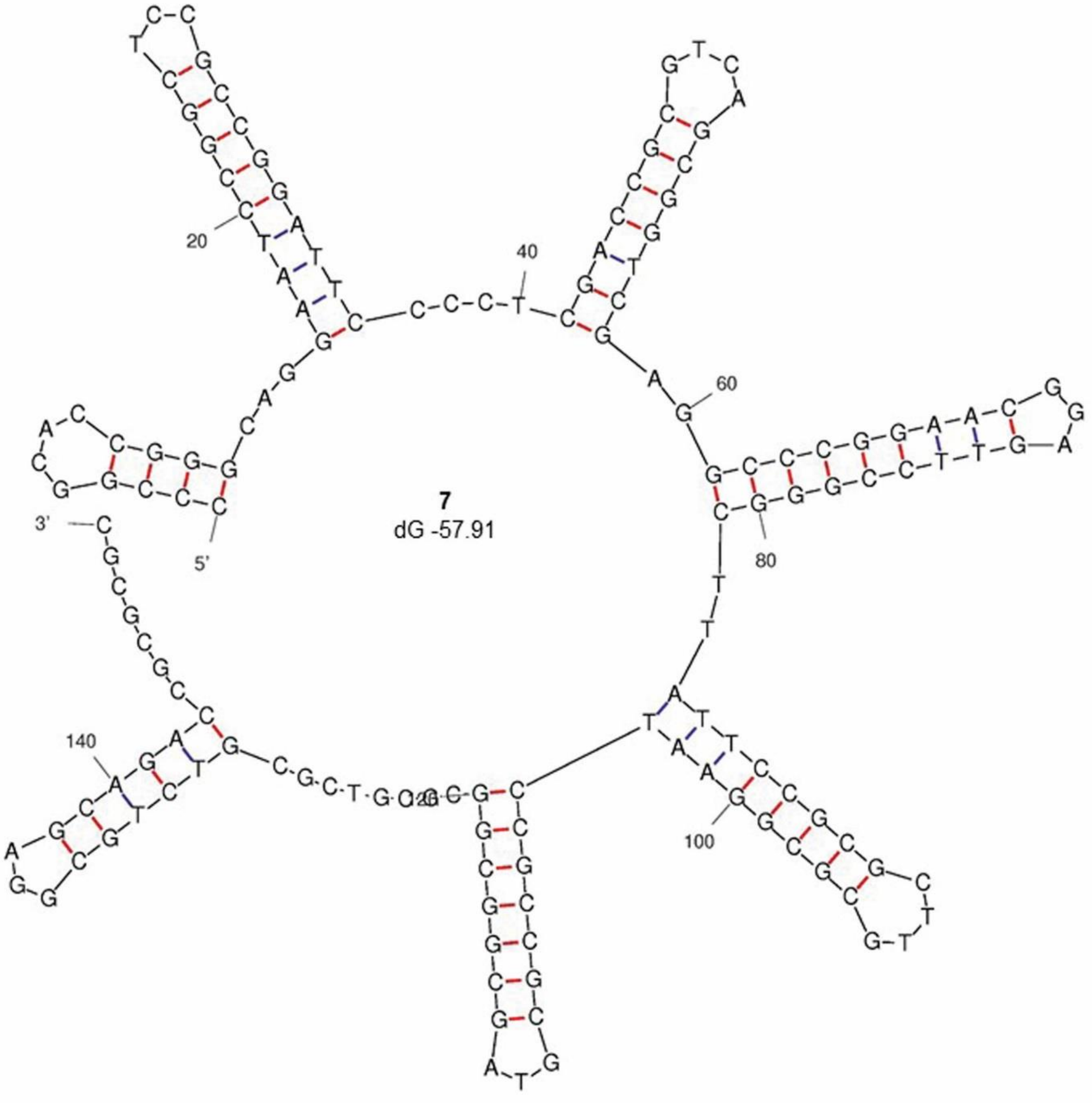
7
dG -57.91

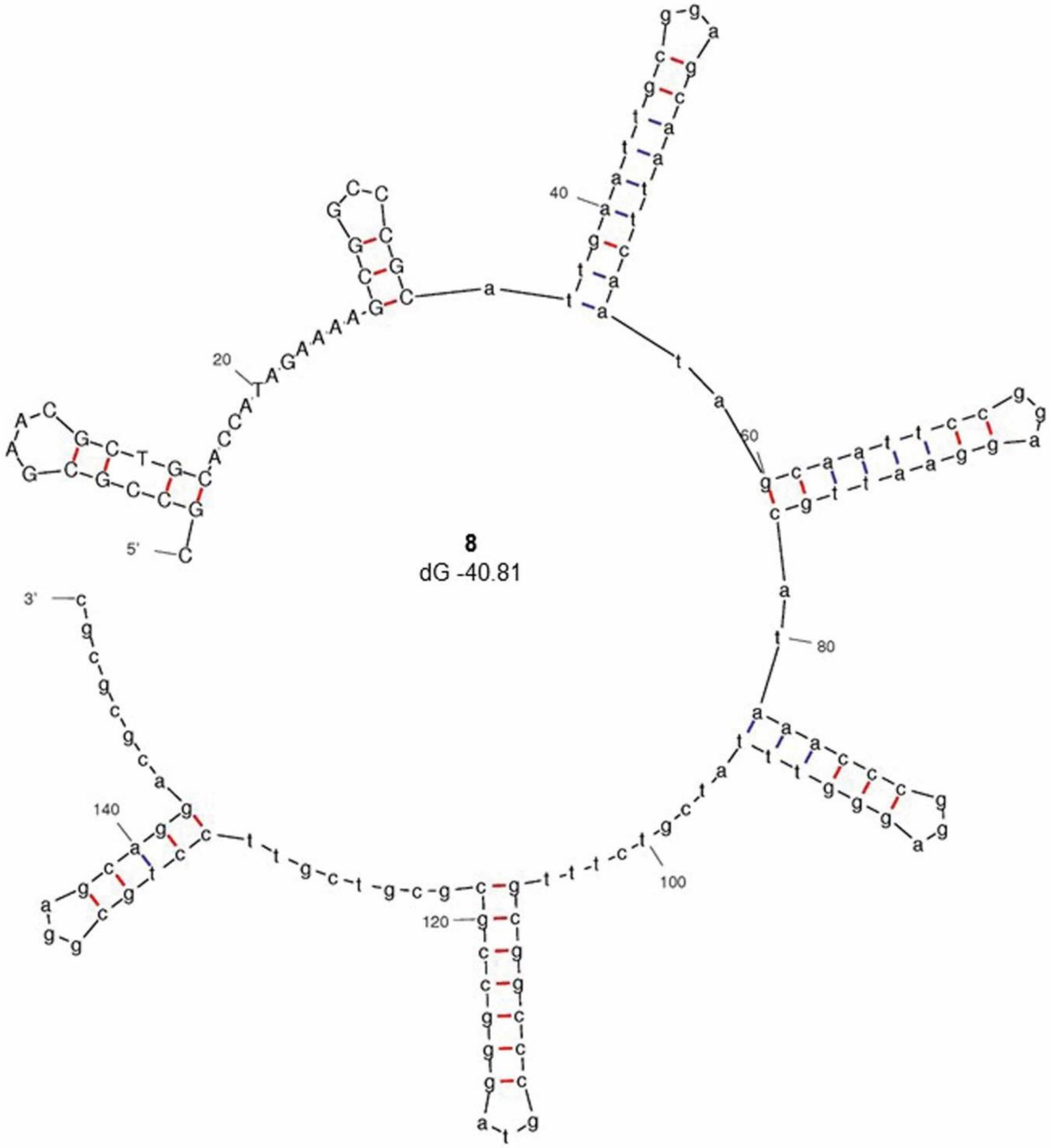
8
dG -40.81
5'
3'
20
40
60
80
100
120
140

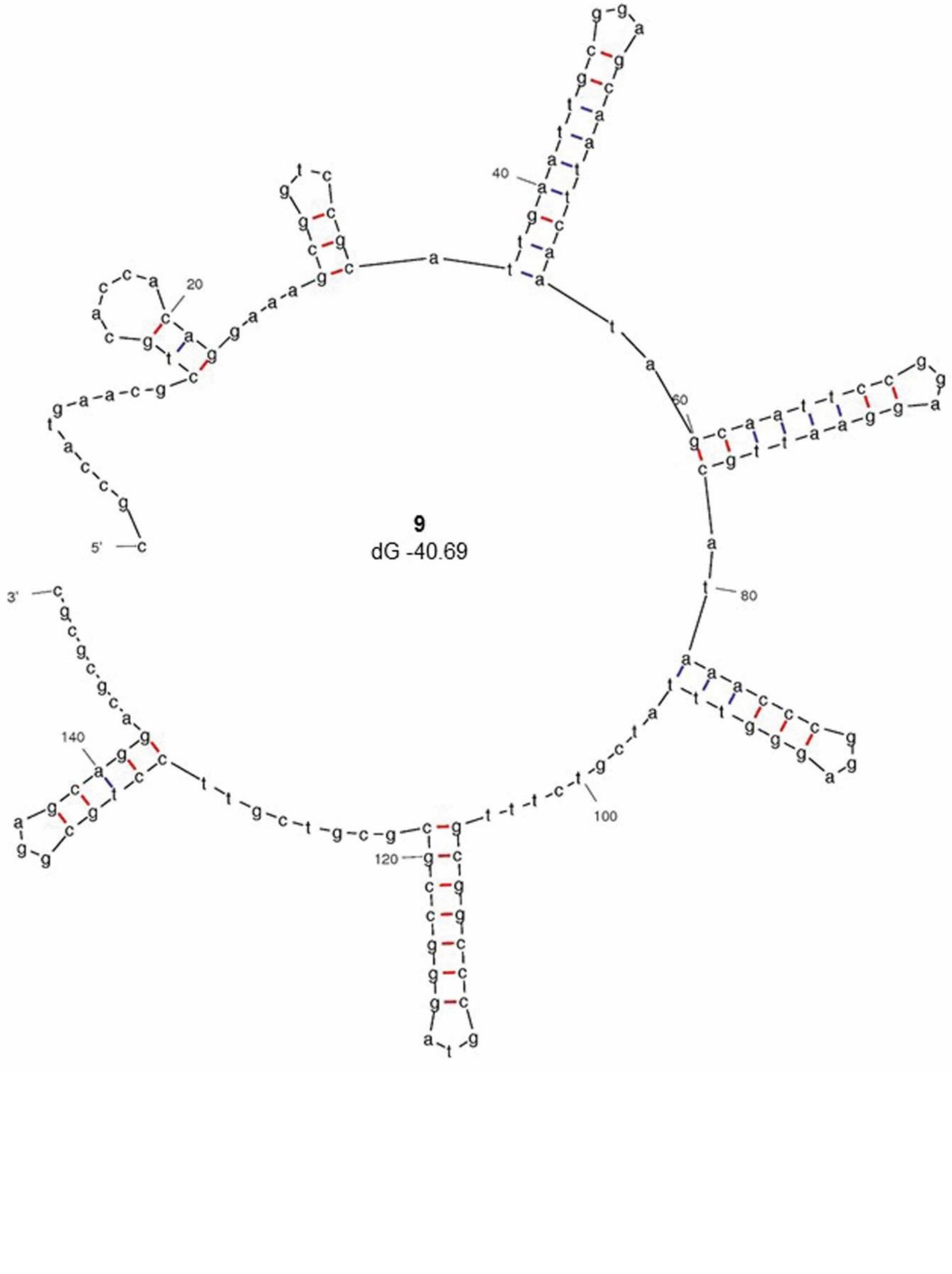
9
dG -40.69
5'
3'
20
40
60
80
100
120
140

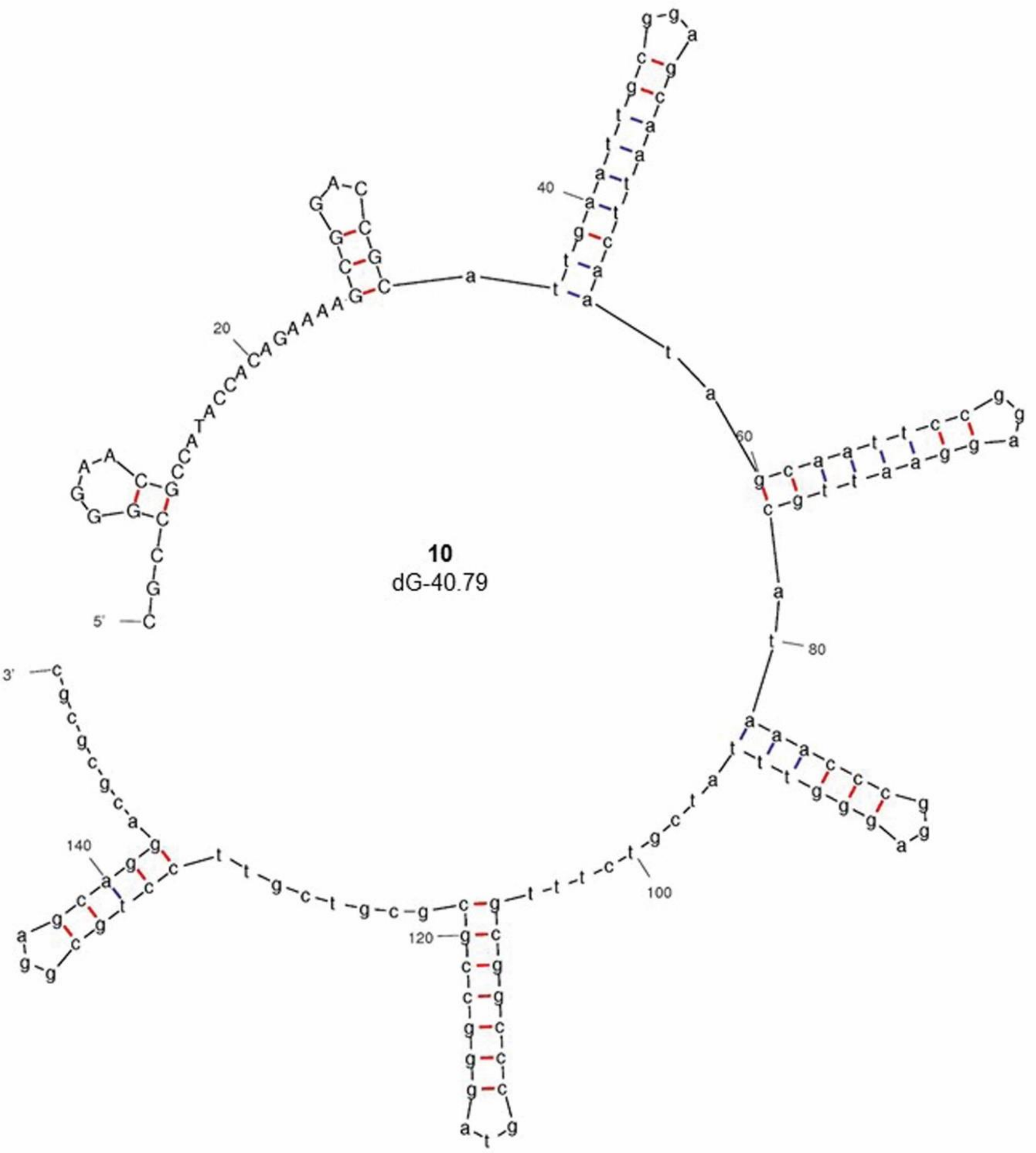

10
dG-40.79
5'
3'
20
40
60
80
100
120
140

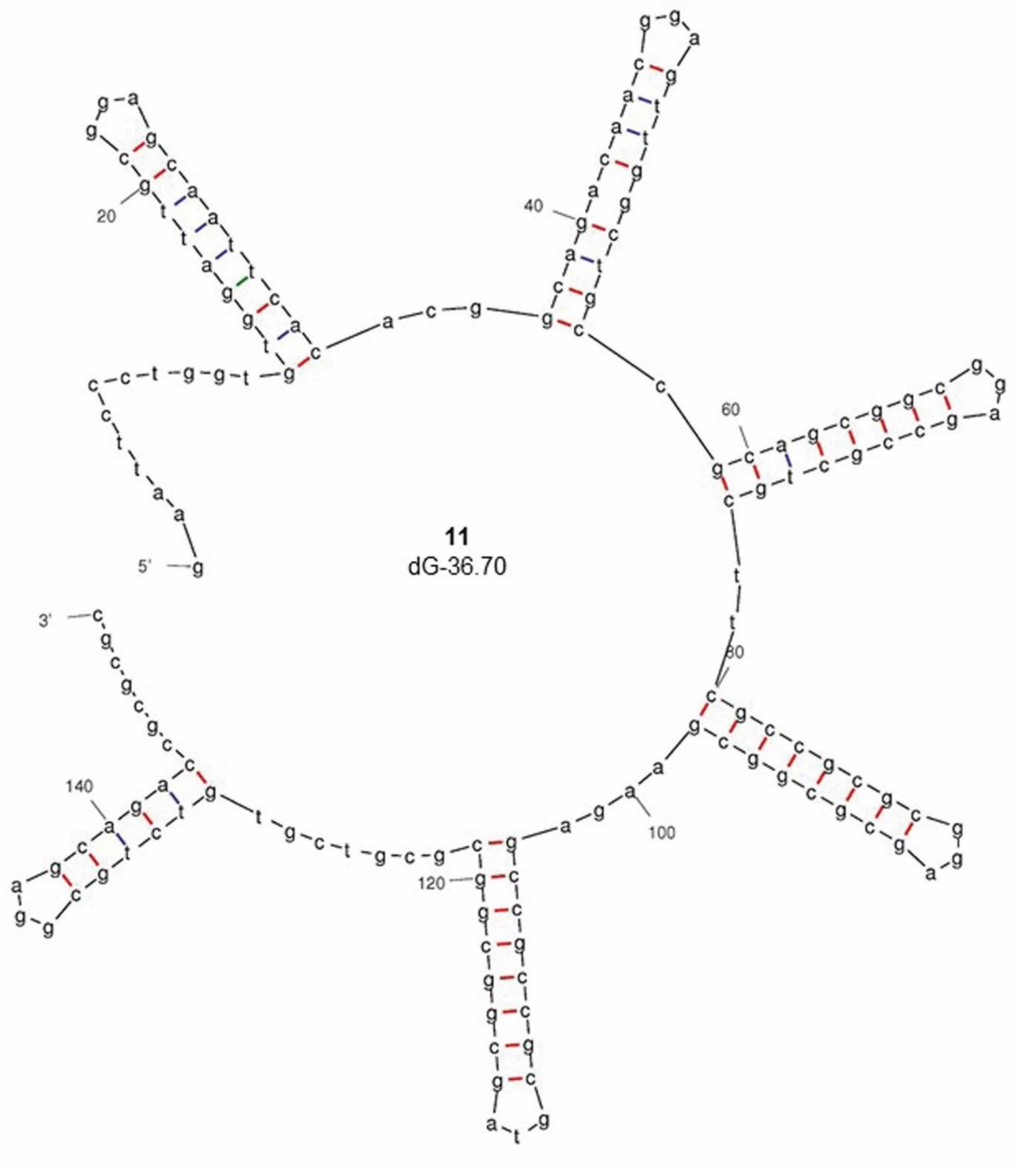
11
dG-36.70
5'
3'
20
40
60
80
100
120
140

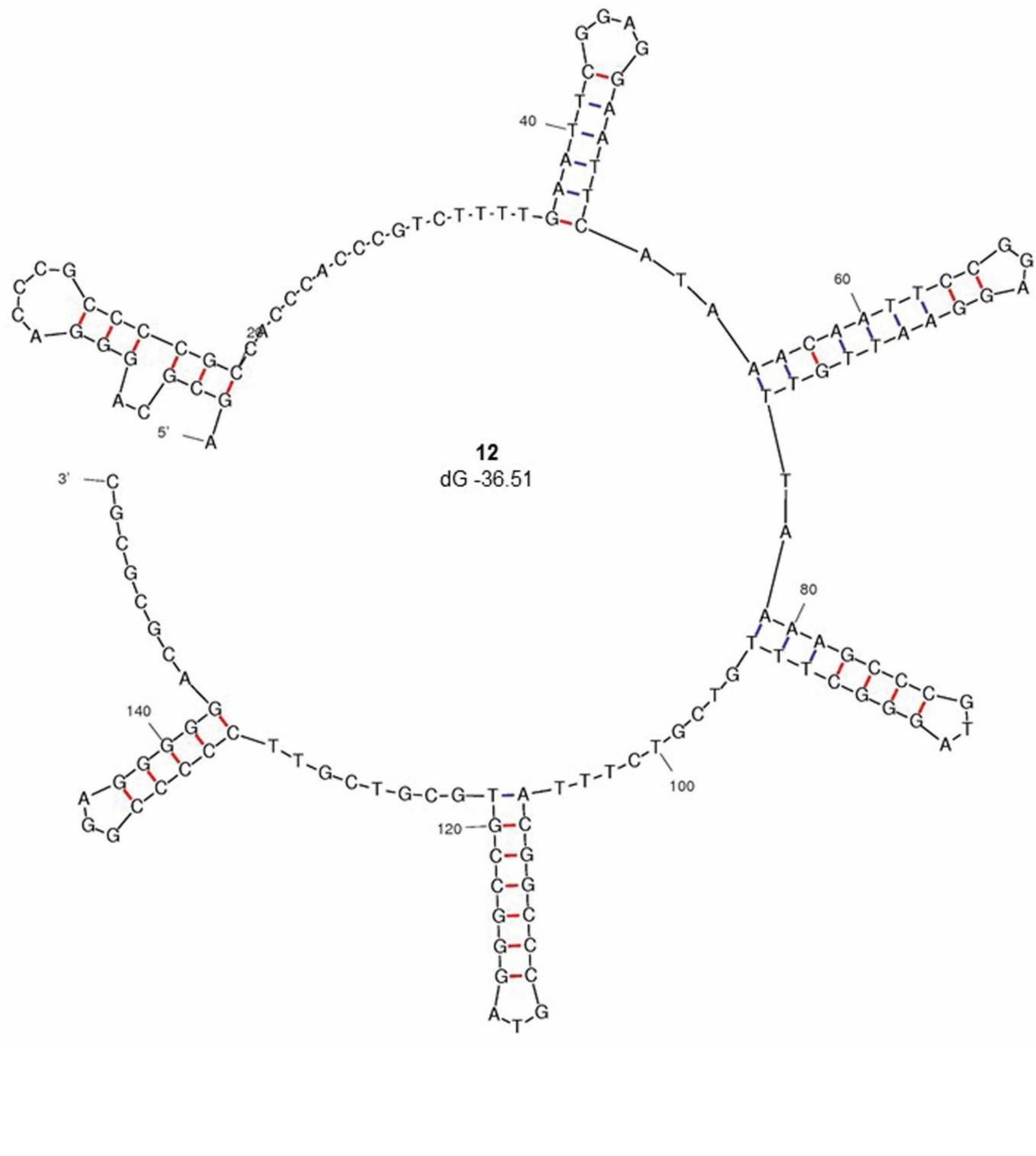
12
dG -36.51
5'
3'
20
40
60
80
100
120
140

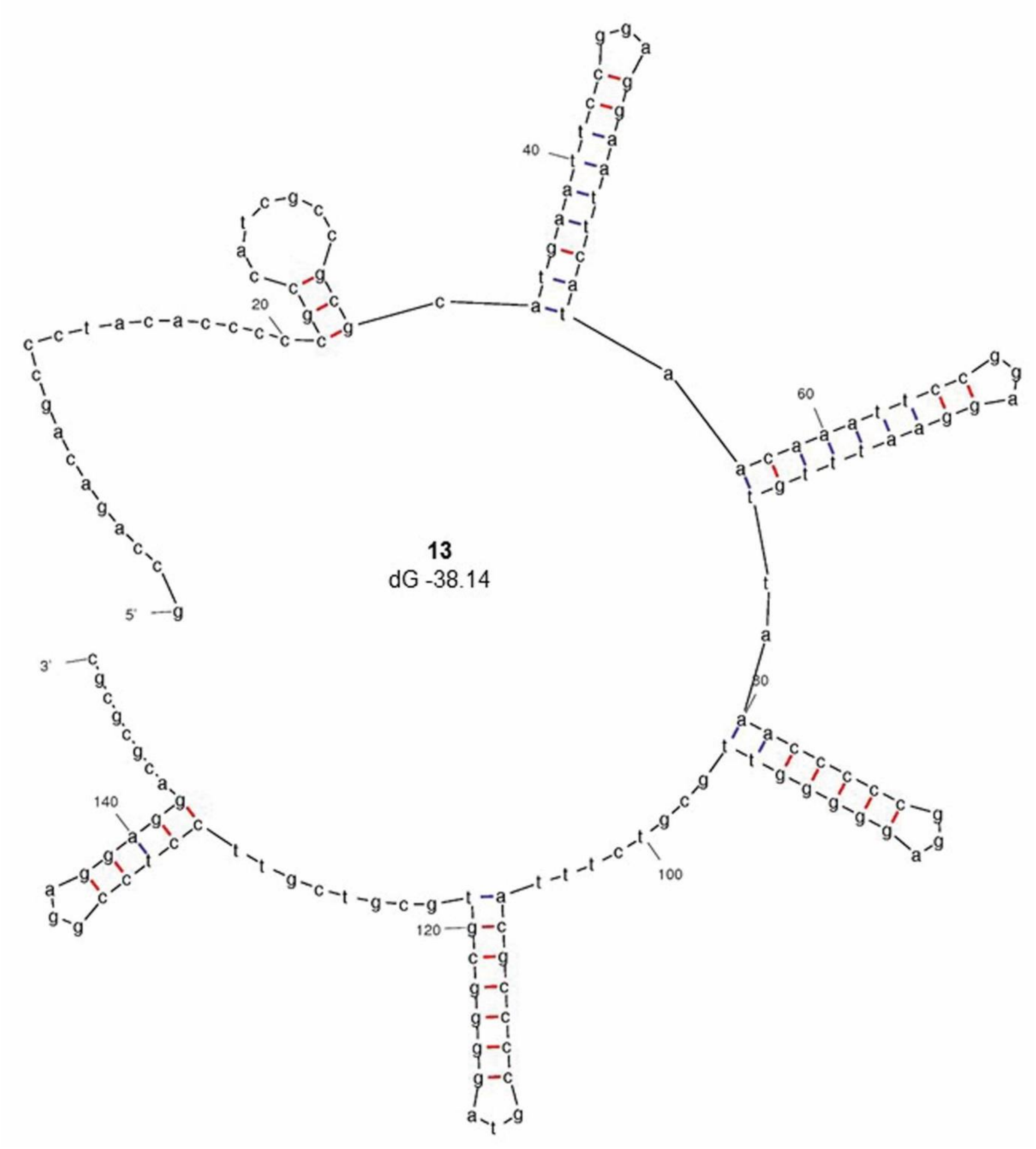

Fig S3. Mfold projections of Sg2247-type telomeres. The terminal 150bp of 3'replicon ends of Sg2247-type telomeres showing stem-loop structures and hairpins (I-VIII) identified using Mfold (7) are displayed where the revised free energies (dG, kJ/mol) were determined using Jacobson-Stockmeyer theory to assign free energies to multi-branch loops. Projections were calculated using default conditions except folding temperature was set at 30°C, Na^{+} concentration of 0.05M and maximum distance between paired bases was set at 20 (8).

Further details of the 13 Sg2247-type telomeres are described in Table S2. 1, *S. albidofavus* J1074 chromosome, accession number NC_020990.1; 2, *Streptomyces* sp. HK1 plasmid pSHK1, EU372836.1; 3, *Streptomyces* sp. 36R-2-1B plasmid pYY8L, GU080325.1; 4, *S. cattleya* NRRL 8057 chromosome, FQ859185.1; 5, *S. cattleya* NRRL 8057 plasmid pSCAT, FQ859184.1; 6, *S. hygroscopicus* subsp. Jinggangensis TL01 plasmid pSHJGH1 right hand end, NC_020894.1; 7, *S. hygroscopicus* subsp. Jinggangensis 5008 plasmid pSHJG1 right hand end, NC_017766.1; 8, *S. rimosus* ATCC10970 chromosome, CP048261.1; 9, *S. rimosus* ATCC10970 plasmid SRP1 left hand end, CP048261.2; 10, *S. rimosus* ATCC10970 plasmid SRP1 right hand end, CP048261.2; 11, *S. pratensis* ATCC 33331 plasmid pSFLAO1, CP002476.1; 12, *S. griseus* 2247 chromosome; 13, *Streptomyces* sp. 769 plasmid pSGZL, CP003988.1. Further details of known streptomycete telomeres are listed in Table S2.

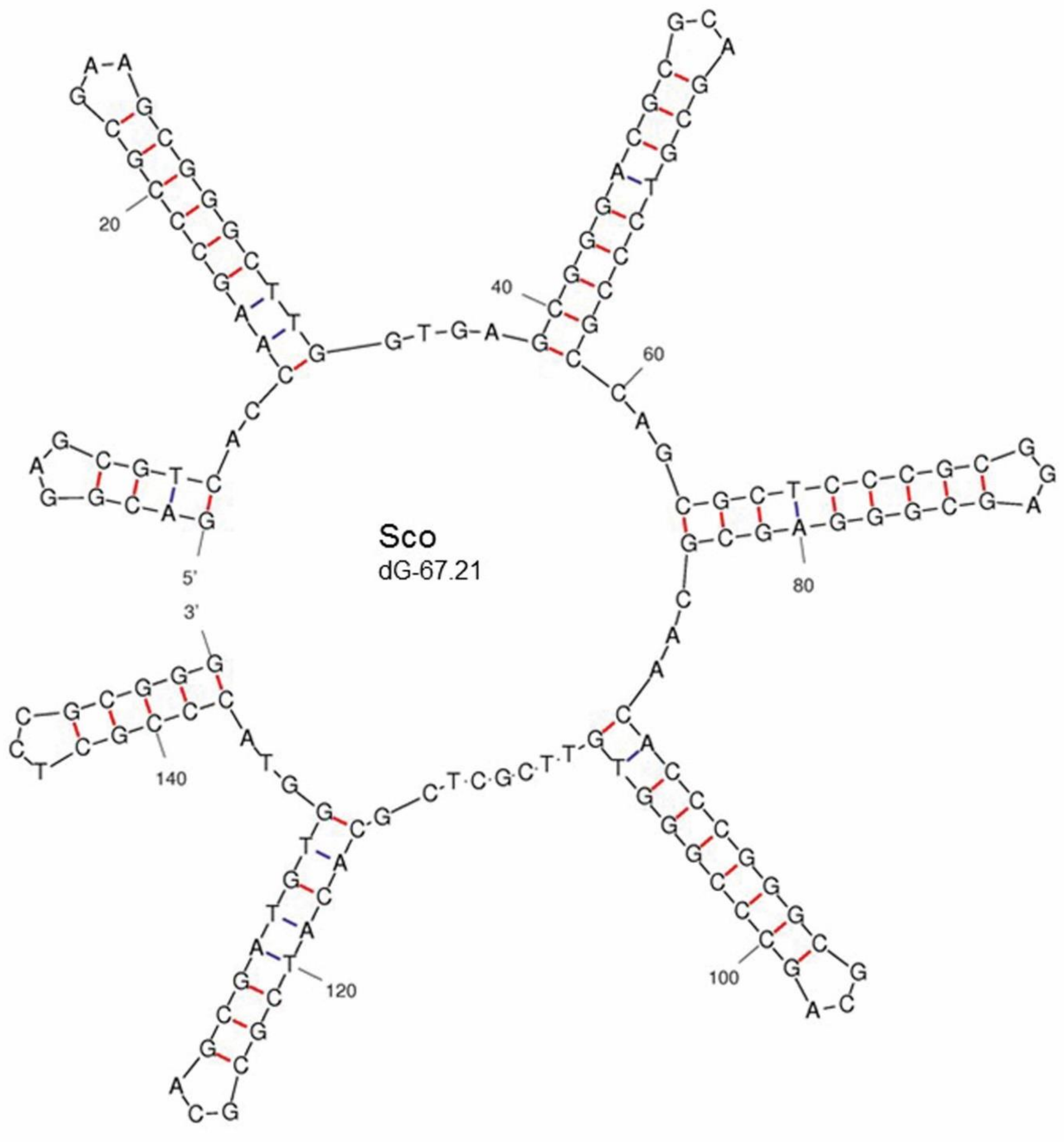
Sco
dG-67.21
5'
3'
20
40
60
80
100
120
140

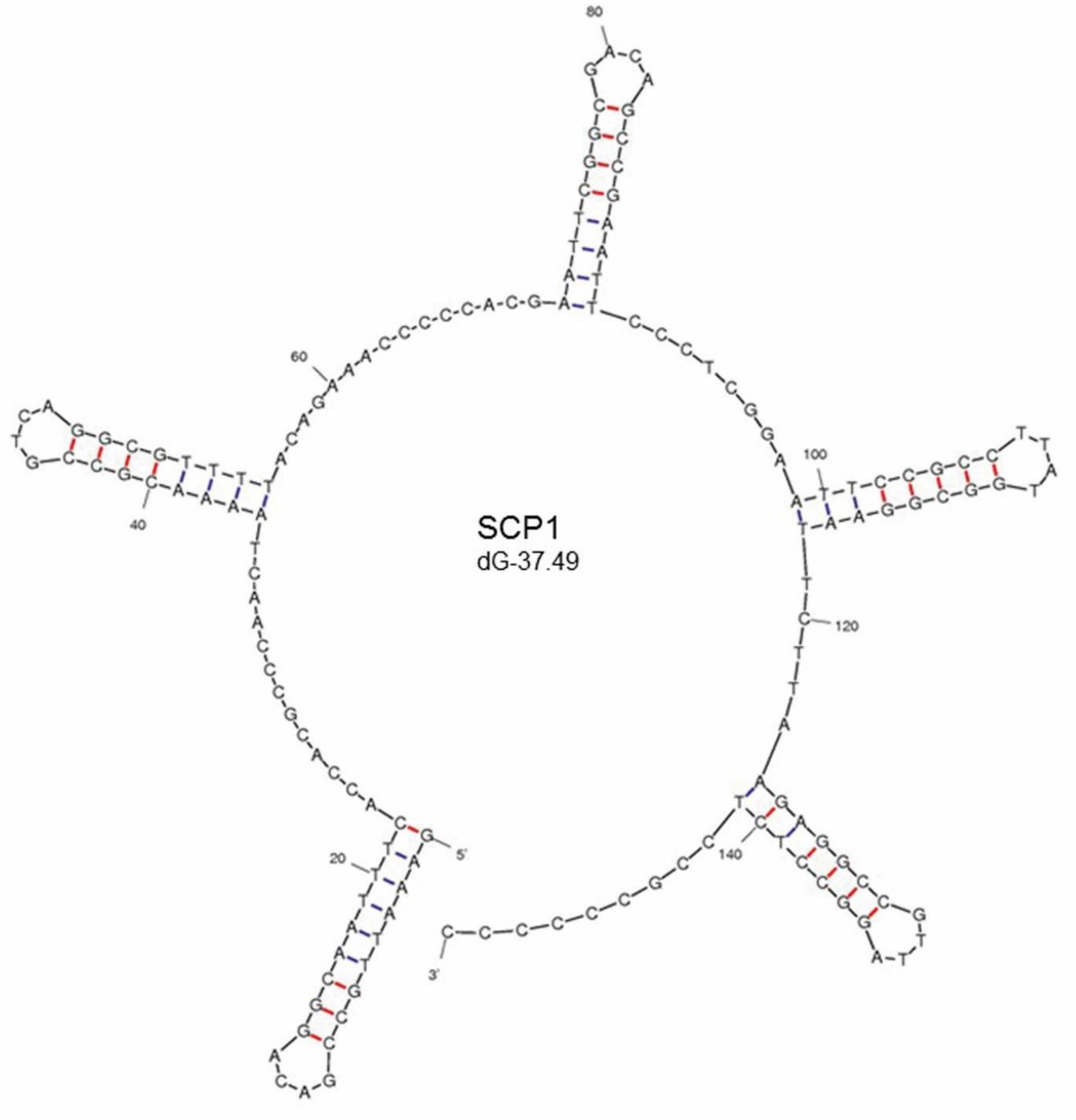
SCP1
dG-37.49
80
60
100
40
120
20
140
5'
3'

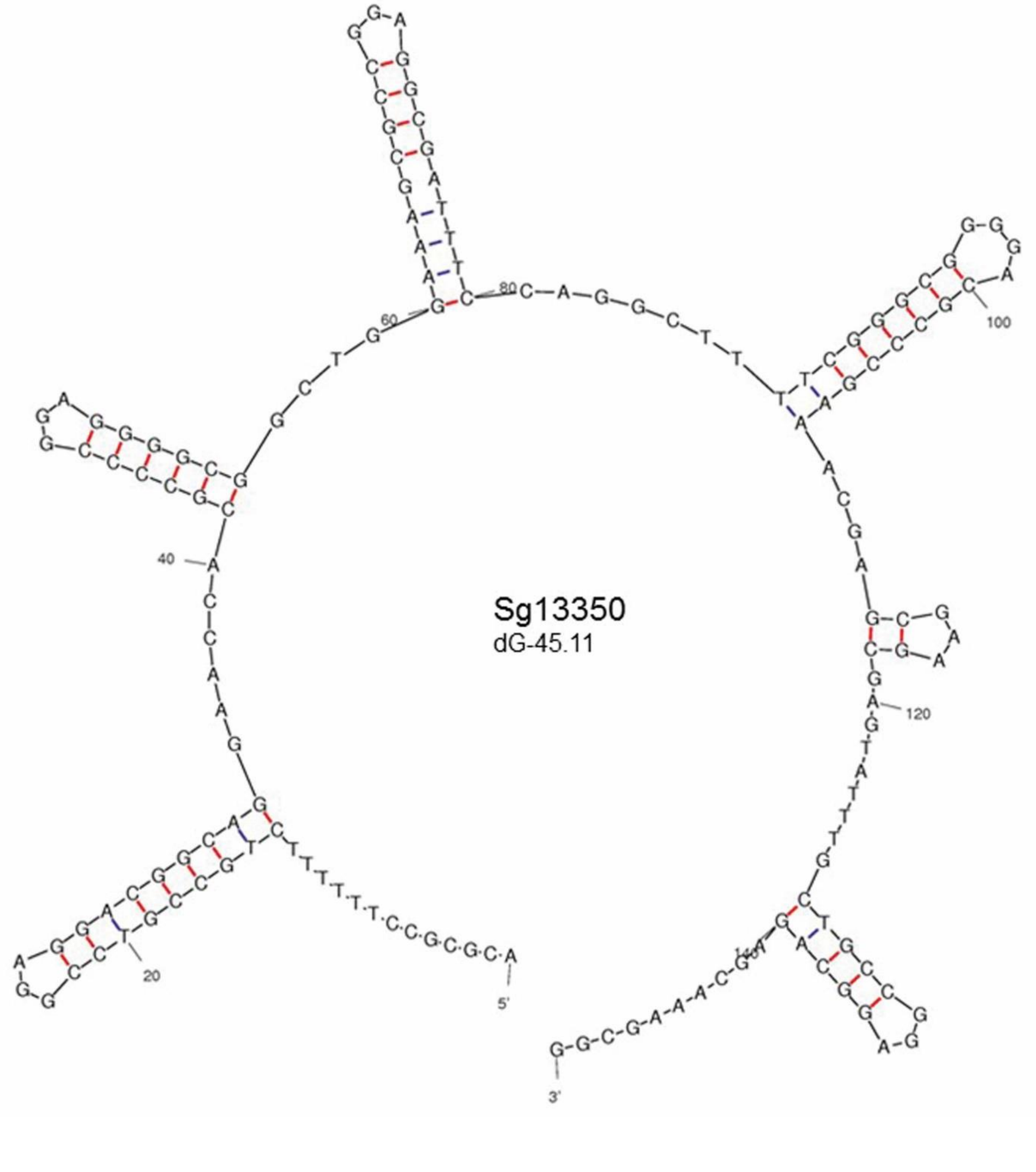
Sg13350
dG-45.11
5'
3'

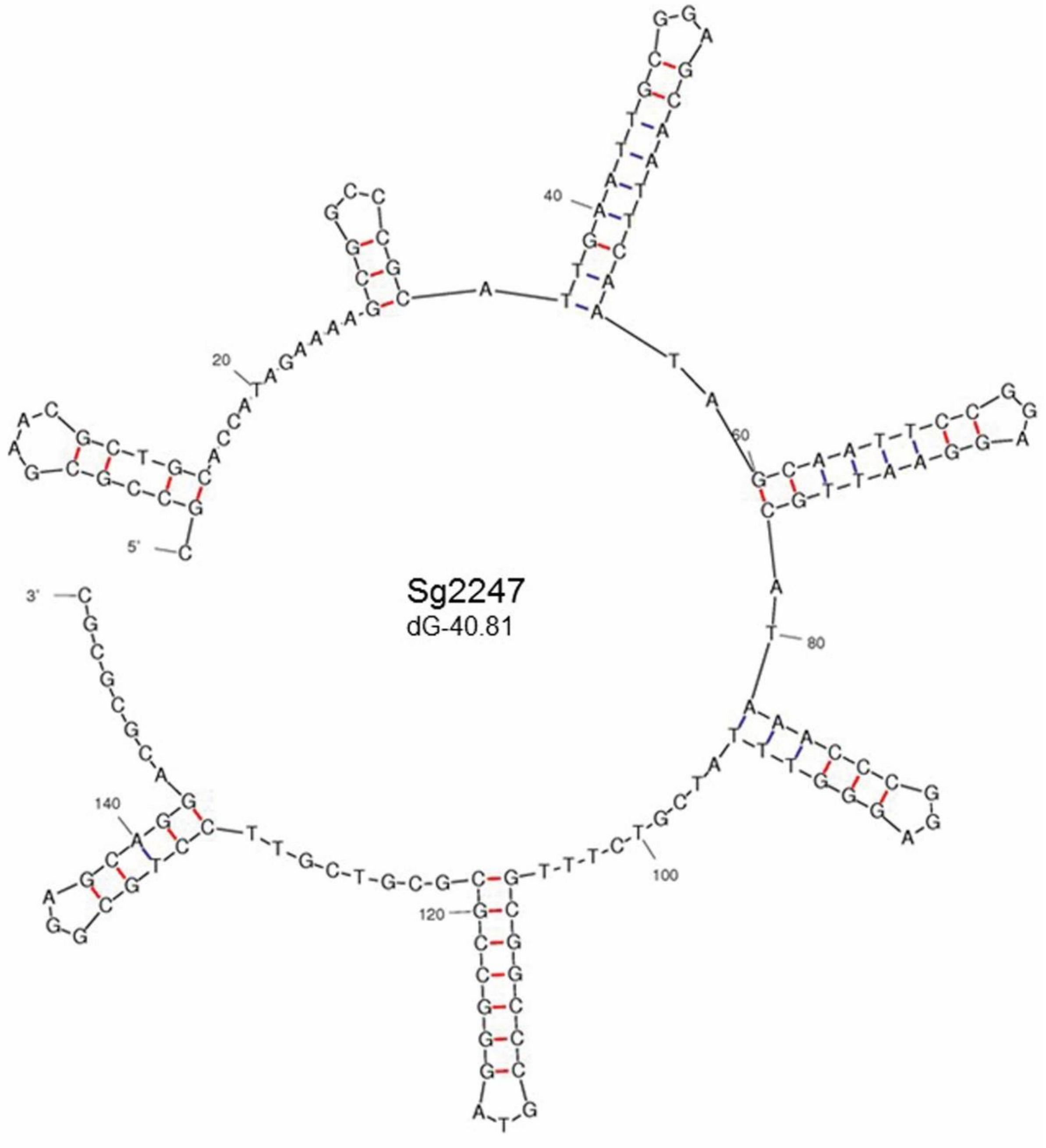
Sg2247
dG-40.81

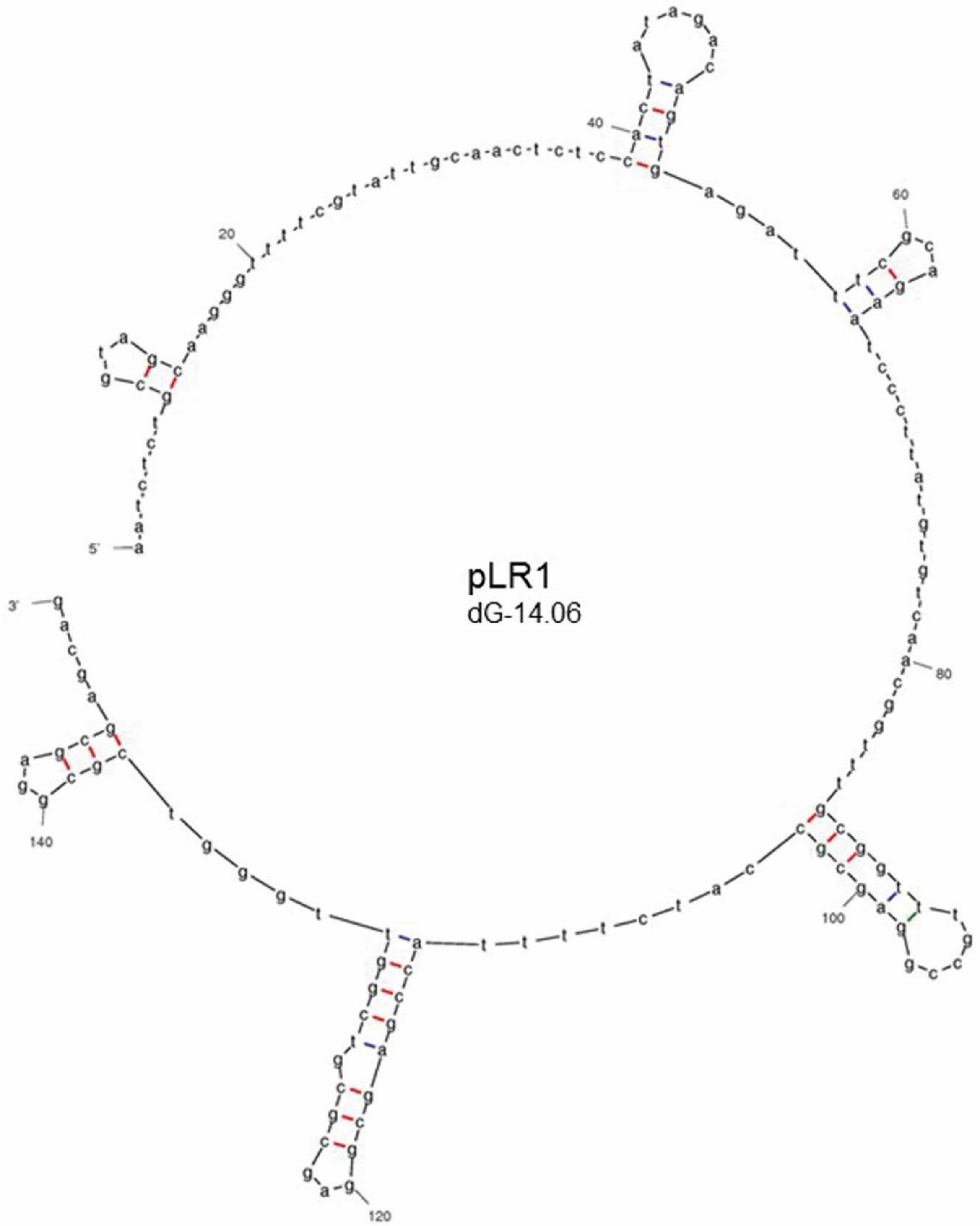
pLR1
dG-14.06
5'
3'
20
40
60
80
100
120
140

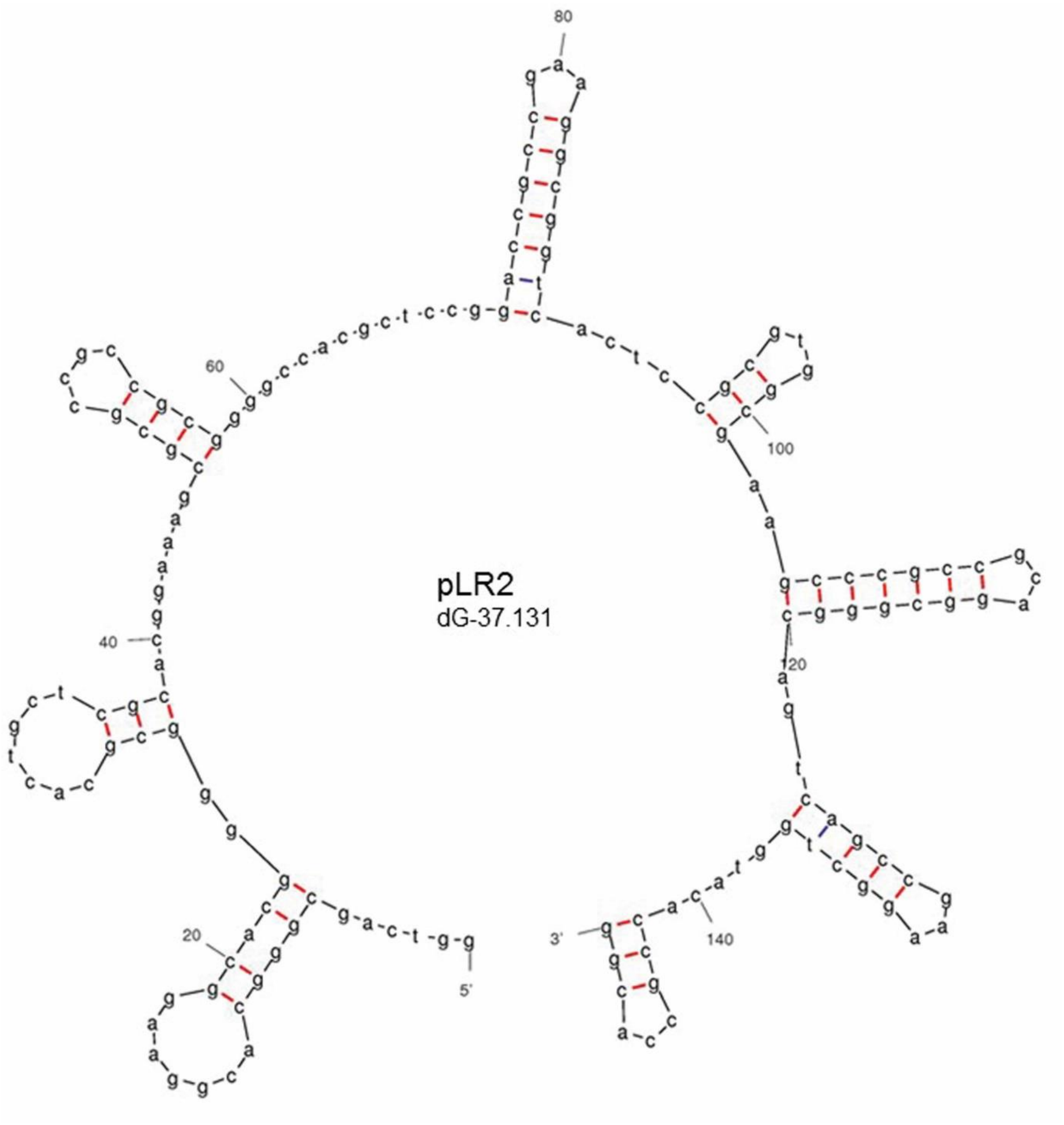

Fig S4. The six known classes of streptomycete telomeres. Mfold projections of the terminal 150 bp of the known classes of streptomycete telomeres showing stem-loop structures (underlined, arrows) and hairpins (I-VIII, red) identified using Mfold (7) are displayed where the revised free energies (dG) were determined using Jacobson-Stockmeyer theory to assign free energies to multi-branch loops. Projections were calculated using default conditions except folding temperature was set at 30°C, Na^+ concentration of 0.05M and maximum distance between paired bases was set at 20 (8). Sco, archetypal end from *S. coelicolor* chromosome (9); SCP1, non-archetypal end from *S. coelicolor* plasmid SCP1 (10); Sg13350, chromosome end from *S. griseus* 13350 chromosome (11); Sg2247, chromosome

end from *S. rimosus* ATCC10970 chromosome (this work); pRL1 end from *Streptomyces* sp. 44030 plasmid pRL1(12); pRL2 end from *Streptomyce*s sp. 440414 plasmid pRL2 (12).

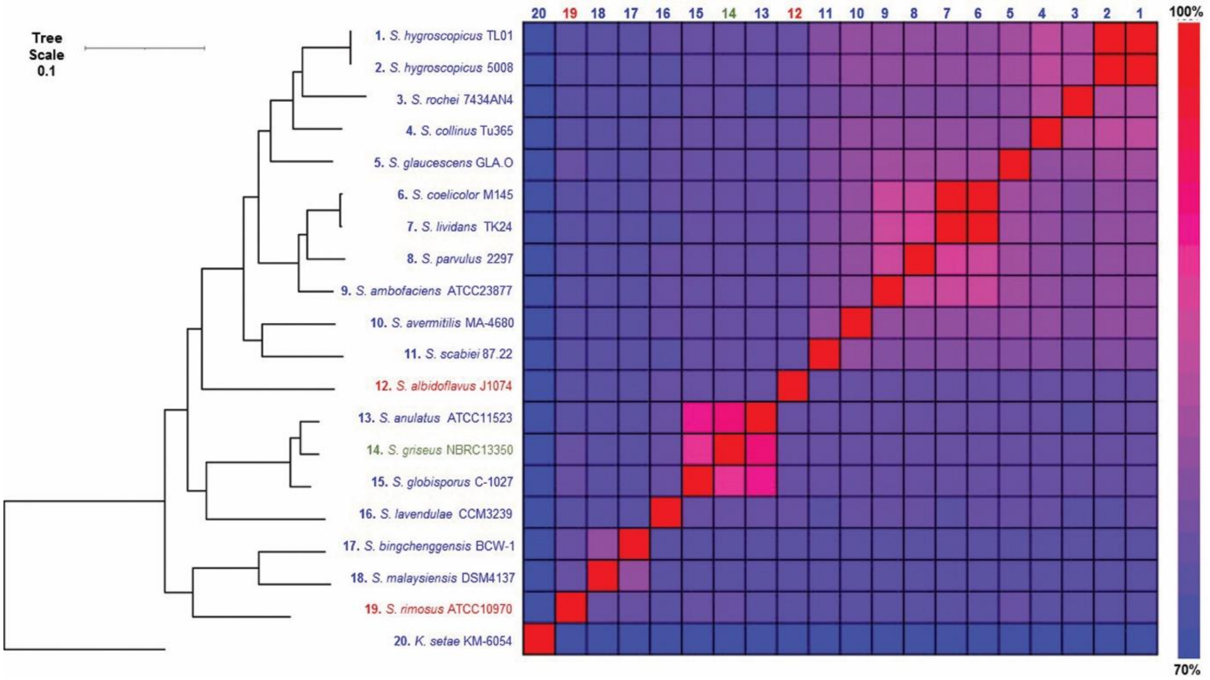

Fig S5. Phylogenetic relationship of the chromosomes from members of the *Streptomycetaceae* with closed genomes.

Twenty closed genomes (Table S2) from the *Streptomyetaceae*, where all replicons were completely sequenced and were either circular or, when linear, were flanked by one of the six classes of streptomycete telomeres, were arranged so that *dnaN* and *dnaA* were coded on the bottom stand and *parA* and *parB* on the top strands*.* The nineteen chromosome sequences from the genus *Streptomyces*, in conjunction with the closed sequence of *K. setae* KM-6054 as an outgroup, were then used to carry out Multi-Locus Sequence Analysis (MLSA) to produce a high-resolution species tree using AutoMLST after a concatenated alignment (13). All branches were supported with boot strap values of 100 except the *S. hygroscopicus*/*S. rochei* node (66) and *S. lividans*/*S. parvulus* node (98). Chromosomes with archetypal chromosomes are listed in blue, Sg2247 in red and Sg13350 in green. The average nucleotide identities (ANI) between the 20 closed S*treptomycetaceae* genomes was calculated using the OrthoANI tool (14) and plotted with the heat map function in R (Ver. 4.0).

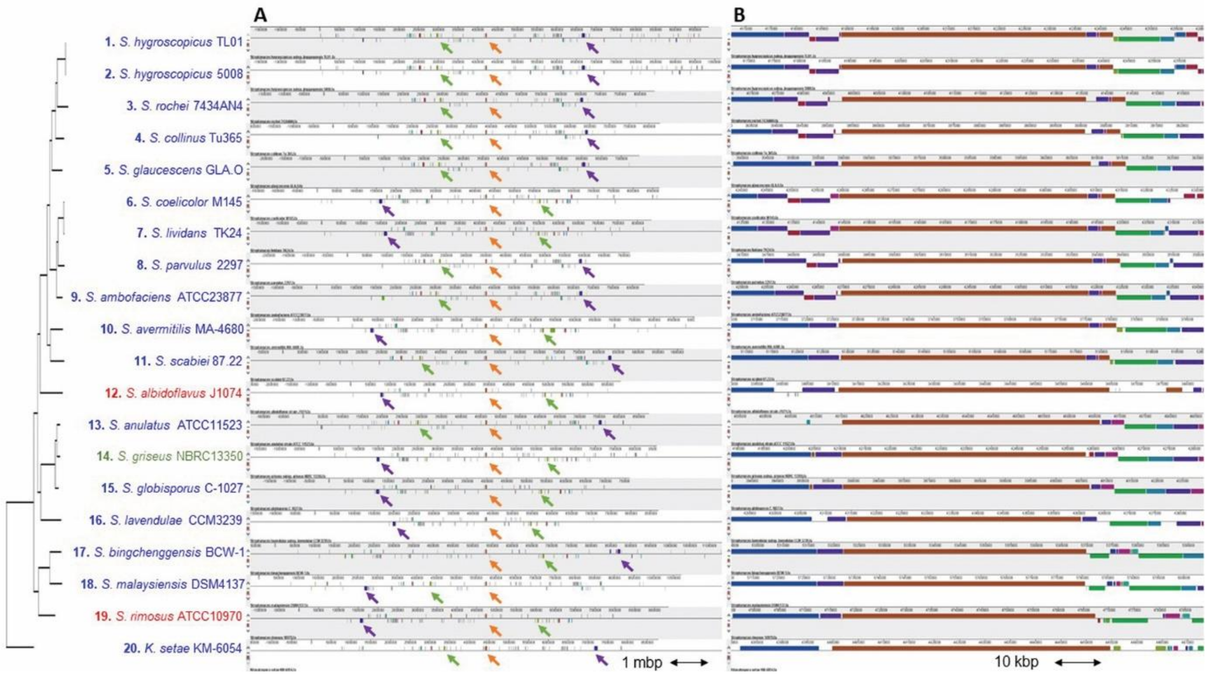

Fig S6. *Streptomycetaceae* chromosomes are centred on a ~50kb origin island.

All 20 closed *Streptomycetaceae* chromosomes were subjected to a progressive alignment in Mauve (15) using default settings. Replicons were centred on the *oriC* region of *S. coelicolor* M145 from *SCO3872-SCO3911* (brown locally collinear block (LCB) (brown arrow)) and displayed as the entire chromosome (A). Representative LCBs (green/purple LCBs/arrows) flanking the origin island showing their location in different replichores from different strains are also displayed. The *oriC* region is also displayed showing the conserved ~50-Kb origin island (B). This brown LCB corresponding to the *oriC* region of all chromosomes is flanked by a tRNA-ile (left) and *dnaB* (right, *SCO3911*) and represents the origin island. Chromosomes with archetypal telomeres are listed in blue, Sg2247 telomeres in red and Sg13350 in green.

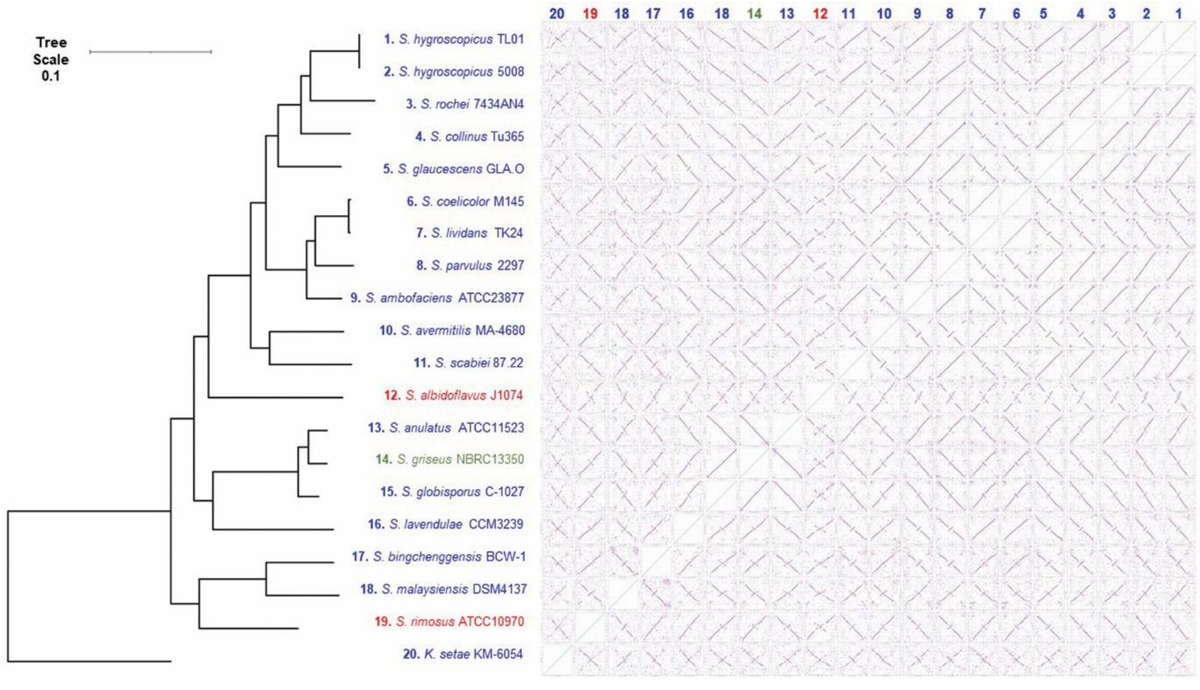

Fig S7. Symmetrical genomic rearrangements in *Streptomycetaceae* chromosomes. Dotplots comparing each of the 20 closed streptomycete chromosomes with each other, organized so that *oriC* regions were syntenous, were determined using Nucmer (16). Reference sequences are displayed in columns (20-1) and query sequences in rows (1-20). Chromosomes with archetypal telomeres are listed in blue, Sg2247 telomeres in red and Sg13350 in green. Individual Nucmer plots of strains around selected nodes are displayed in more detail in Fig 5.

Name	Sequence (5’-3’)
Lis-SrimC-iPCR-1	ttgcaaaaatcgtcggttgggagtcggt
Lis-SrimC-iPCR-2	catcacgctgcatcagttttggagaaacgt
Lis-SrimP-iPCR-L1	atgacgcgattttccgaggcctttccgta
Lis-SrimP-iPCR-L2	ccggagggtttattatctttgcggcccgta
Lis-SrimP-iPCR-R1	gccttgcatcatgctgcatcagtttcgggaaaa
Lis-SrimP-iPCR-R2	agaacaatgcgggaaagtcgagcgtgatc

Table S1. iPCR primers for recovery of *S. rimosus* chromosomal and left & right plasmid telomeres.

Table S2 Known and predicted *Streptomycetaceae* telomeres

Telomeres were identified on the basis of similarity with the six reported classes of streptomycete telomeres (archetypal, non-archetypal (SCP1), Sg2247, Sg13350, plR1 and pLR2. The sequences are displayed in Fig. 4 were used as representatives of each telomeric class. The terminal 150bp or the sequence corresponding to the two terminal stem loop structures of each telomere class were used as query sequences in Blastn (NCBI). Hits were manually curated; those sequences that were either physically recovered and confirmed through Sanger sequencing or were found at the end of replicons were included as members of the four different telomeric classes. Telomeres were further sub-divided on the basis of their location (chromosome or linear plasmid; location at one or both ends of replicon) or means of identification (whole genome sequencing, physical recovery and traditional Sanger sequencing). Accession numbers for all sequences are displayed except that from *S. griseus* 2247 (not present in NCBI and only exists as a Figure in the article describing its recovery and characterisation (17)).

Table S3 Closed genomes from the *Streptomycetatceae*

Organisms with closed genomes of the *Streptomyetaceae* were defined as such on the basis that they are described as “complete” in NCBI and that all linear replicons found in that organism were flanked by known members of the six telomeric classes (Fig. 4) Archetypal (Sco), non-archetypal (SCP1), Sg13350, Sg2247, pLR1, pLR2. On this basis, although at time of writing 223 complete *Streptomycetaceae* genomes are listed in NCBI, only 20 met our criteria as closed. Some organisms met some criteria for inclusion in our list of closed genomes, such as the chromosomes of *Streptomyces* sp. ETH9427 and *Streptomyces clavuligerus* F1D-5. However these organisms were not included in our closed list as these strains carried linear plasmids without recognizable telomeres. The length of the Terminal Inverted repeats at the ends of each replicon were determined by aligning each end of a replicon to the other end. This was done using the align sequence tool in Snapgene^{TM} and

allowed the identification of both perfect and imperfect repeat regions. Orthologues of genes from these 20 strains that encoded the proteins Tap (SCO7733, Tpg (SCO7734), TtrA (SCO0002), DnaN (SCO3878), DnaA (SCO3879), ParA (SCO3886), ParB (SCO3887) and RecA (SCO5769) were determined using BlastP (NCBI) with the proteins from *S . coelicolor* A3(2) as queries; only hits with at least 25% identity and coverage were included.

Table S4 Origin islands and *parS* sites from the *Streptomycetaceae*

The location of predicted *parS* sites with respect to origin islands on the chromosomes of the 20 closed *Streptomycetaceae* genomes were determined by searching the 20 these genomes using the consensus matrix for bacterial *parS* sites (18). This was done by performing a ClustalW alignment of the 1030 predicted *parS* sited previously identified (18). A consensus matrix was then constructed in Weblogo (http://weblogo.threeplusone.com/create.cgi) (19) and used to interrogate the 20 closed *Streptomyetaceae* genomes using matrix scan in the Regulatory Sequence Analysis Tools suite (RSAT) (http://embnet.ccg.unam.mx/rsat//matrix-scan-quick_form.cgi) (20). For this analysis the Threshold weight score was set at >15 (18). This allowed predicted *parS* sites to be mapped onto the chromosome and their locations determined with respect to the origin island and in relation to *oriC*. Each location was also normalised to genome size by expressing the site location as a percentage of the size of the chromosome. In *S. coelicolor* the origin island contains the following genes. tRNA-ile, *SCO3872, SCO3873* (*gyrA*), *SCO3874* (*gyrB*), *SCO3875, SCO3876* (*recF*), *SCO3877, SCO3878* (*dnaN*), *oriC, SCO3879* (*dnaA*), *SCO3880* (*rpmH*), *SCO3881* (*rnpA*), *SCO3882, SCO3883* (*yidC*), *SCO3884, SCO3885* (*gidB*), *SCO3886* (*parA*), *SCO3887* (*parB*), *SCO3888, SCO3889* (*trxA*), *SCO3890* (*trxB*), *SCO3891, SCO3892* (*sigT*), *SCO3893, SCO3894, SCO3895, SCO3896, SCO3897, SCO3898, SCO3899, SCO3900, SCO3901, SCO3902, SCO3903, SCO3904, SCO3905, SCO3906* (*rpsF*), *SCO3907 (ssbA), SCO3908* (*rpsR*), *SCO3909 (rplI), SCO3910, SCO3911 (dnaB).* In *S. coelicolor oriC* lies between *SCO3878* (*dnaN*) and *SCO3879* (*dnaA*), after arranging all chromosomes so that these genes were transcribed in right to left direction, the last base of *dnaA* from all genomes was selected as the location of *oriC*. The locations of these sites within the core region of the chromosome and with respect to *oriC* are shown in Fig. 6. The location and sequence of putative *parS* sites across all 20 closed genomes is provided in Supplementary File S5.

1. Gravius B, Glocker D, Pigac J, Pandža K, Hranueli D, Cullum J. The 387 kb linear plasmid pPZG101 of *Streptomyces rimosus* and its interactions with the chromosome. Microbiology. 1994;140(9):2271-7.
2. Thoma L, Vollmer B, Muth G. Fluorescence microscopy of *Streptomyces* conjugation suggests DNA-transfer at the lateral walls and reveals the spreading of the plasmid in the recipient mycelium. Environ Microbiol. 2016;18(2):598-608.
3. Pethick FE, Macfadyen AC, Tang Z, Sangal V, Liu TT, Chu J, et al. Draft Genome Sequence of the Oxytetracycline-Producing Bacterium *Streptomyces rimosus* ATCC 10970. Genome announcements. 2013;1(2):e0006313.
4. Langmead B, Trapnell C, Pop M, Salzberg SL. Ultrafast and memory-efficient alignment of short DNA sequences to the human genome. Genome biology. 2009;10(3):R25.

5. Walker BJ, Abeel T, Shea T, Priest M, Abouelliel A, Sakthikumar S, et al. Pilon: an integrated tool for comprehensive microbial variant detection and genome assembly improvement. PloS one. 2014;9(11):e112963.
6. Fan Y, Dai Y, Cheng Q, Zhang G, Zhang D, Fang P, et al. A self-ligation method for PCR-sequencing the telomeres of *Streptomyces* and *Mycobacterium* linear replicons. Journal of microbiological methods. 2012;90(2):105-7.
7. Zuker M. Mfold web server for nucleic acid folding and hybridization prediction. Nucleic acids research. 2003;31(13):3406-15.
8. Yang CC, Tseng SM, Pan HY, Huang CH, Chen CW. Telomere associated primase Tap repairs truncated telomeres of *Streptomyces*. Nucleic acids research. 2017;45(10):5838-49.
9. Bentley SD, Chater KF, Cerdeno-Tarraga AM, Challis GL, Thomson NR, James KD. Complete genome sequence of the model actinomycete Streptomyces coelicolor A3(2). Nature. 2002;417.
10. Huang CH, Tsai HH, Tsay YG, Chien YN, Wang SL, Cheng MY, et al. The telomere system of the *Streptomyces* linear plasmid SCP1 represents a novel class. Molecular microbiology. 2007;63(6):1710-8.
11. Ohnishi Y, Ishikawa J, Hara H, Suzuki H, Ikenoya M, Ikeda H, et al. Genome sequence of the streptomycin-producing microorganism *Streptomyces griseus* IFO 13350. Journal of bacteriology. 2008;190(11):4050-60.
12. Zhang R, Yang Y, Fang P, Jiang C, Xu L, Zhu Y, et al. Diversity of telomere palindromic sequences and replication genes among *Streptomyces* linear plasmids. Applied and environmental microbiology. 2006;72(9):5728-33.
13. Alanjary M, Steinke K, Ziemert N. AutoMLST: an automated web server for generating multi-locus species trees highlighting natural product potential. Nucleic acids research. 2019;47(W1):W276-W82.
14. Lee I, Ouk Kim Y, Park SC, Chun J. OrthoANI: An improved algorithm and software for calculating average nucleotide identity. Int J Syst Evol Microbiol. 2016;66(2):1100-3.
15. Darling AE, Mau B, Perna NT. ProgressiveMauve: multiple genome alignment with gene gain, loss and rearrangement. PloS one. 2010;5(6):e11147.
16. Marcais G, Delcher AL, Phillippy AM, Coston R, Salzberg SL, Zimin A. MUMmer4: A fast and versatile genome alignment system. PLoS computational biology. 2018;14(1):e1005944.
17. Goshi K, Uchida T, Lezhava A, Yamasaki M, Hiratsu K, Shinkawa H, et al. Cloning and analysis of the telomere and terminal inverted repeat of the linear chromosome of *Streptomyces griseus*. Journal of bacteriology. 2002;184(12):3411-5.
18. Livny J, Yamaichi Y, Waldor MK. Distribution of centromere-like *parS* sites in bacteria: insights from comparative genomics. Journal of bacteriology. 2007;189(23):8693-703.
19. Crooks GE, Hon G, Chandonia J-M, Brenner SE. WebLogo: A Sequence Logo Generator. Genome research. 2004;14(6):1188-90.
20. Turatsinze J-V, Thomas-Chollier M, Defrance M, van Helden J. Using RSAT to scan genome sequences for transcription factor binding sites and cis-regulatory modules. Nature Protocols. 2008;3(10):1578-88.
